# *Cytospora* and *Diaporthe* Species Associated With Hazelnut Canker and Dieback in Beijing, China

**DOI:** 10.3389/fcimb.2021.664366

**Published:** 2021-08-02

**Authors:** Hong Gao, Meng Pan, Chengming Tian, Xinlei Fan

**Affiliations:** The Key Laboratory for Silviculture and Conservation of Ministry of Education, Beijing Forestry University, Beijing, China

**Keywords:** Ascomycota, biological characterization, Diaporthales, mycelial growth, phylogeny, taxonomy

## Abstract

Hazelnut (*Corylus heterophylla* Fisch.) is an important nut crop in China but has been declining owing to the destructive effects of fungal branch canker and dieback. The identification and management of these pathogens are difficult because of the lack of attention to branch canker, insufficient understanding of phylogenetic, and overlapping morphological characteristics of the pathogens. In total, 51 strains were isolated from Chinese wild hazelnut in this study, and three species of *Cytospora* and two of *Diaporthe* were identified through morphological observation and multi-locus phylogenetic analyses (ITS, *act*, *rpb2*, *tef1-α*, and *tub2* for *Cytospora*; ITS, *cal*, *his3*, *tef1-α*, and *tub2* for *Diaporthe*). Three new species, *Cytospora corylina*, *C. curvispora*, and *Diaporthe corylicola*, and two known species, *Cytospora leucostoma* and *Diaporthe eres*, grew at 5–30°C and a pH of 3.0–11.0, with optimum growth at approximately 25°C and pH 4.0–7.0. Additionally, the effects of six carbon sources on mycelial growth were investigated. This study explored the main pathogenic fungi species of *Corylus heterophylla*, completed the corresponding database of pathogenic fungi information, and clarified their biological characteristics. Moreover, the results of this study provided a theoretical basis for *Corylus heterophylla* disease management and prevention in China.

## Introduction

Branch canker and dieback are important forest diseases caused by fungal pathogens in the phylum Ascomycota, especially those in the genera *Cytospora* (Cytosporaceae, Diaporthales) and *Diaporthe* (Diaporthecae, Diaporthales) (Adams et al., 2005; Rossman et al., 2007; Gomes et al., 2013; Udayanga et al., 2015; Fan et al., 2020). In total, 672 species epithets of *Cytospora* have been recorded in the Index Fungorum (April 2021; www.indexfungorum.org/). Recent studies reported that approximately 150 species of *Cytospora* caused branch canker and dieback on more than 130 woody host species (Spielman, 1985; Adams et al., 2002; Adams et al., 2005; Kirk et al., 2008; Fan et al., 2014a; Fan et al., 2014b; Fan et al., 2020; Pan et al., 2020; Pan et al., 2021). More than 1,137 species epithets of *Diaporthe* have been enumerated in the Index Fungorum (April 2021; www.indexfungorum.org/), and over 200 species have been accepted recently (Udayanga et al., 2011; Dissanayake et al., 2017; Yang et al., 2020). These species are pathogenic, endophytic, or saprobic to a wide range of plant hosts (Adams et al., 2002; Adams et al., 2005; Udayanga et al., 2011; Fan et al., 2020). Some of these infect the stems, branches, twigs, and even roots of many commercial plants, and cause necrotic damage to young tissues, canker and dieback on branches, and, ultimately, the death of the host, resulting in serious economic losses (Fotouhifar et al., 2010; Udayanga et al., 2011; Udayanga et al., 2012a; Udayanga et al., 2012b; Guerrero and Pérez, 2013b). Symptoms of infected branches include fruiting bodies that immersed in the bark and erupted through the bark surface when mature; however, symptoms are not always the same (Santos et al., 2010; Jiang et al., 2020; Pan et al., 2020). Previous studies on species identification were conducted based on host affiliation and morphology, but different species can infect one host, and single species can infect multiple hosts (Adams et al., 2005; Yang et al., 2020; Zhu et al., 2020). Thus, accurate species identification requires polyphasic approaches based on ecology, additional morphological observations, and multi-locus phylogeny analyses (Harrington and Rizzo, 1999; Mostert et al., 2001; Adams et al., 2002; Adams et al., 2005; Udayanga et al., 2014a; Udayanga et al., 2014b; Du et al., 2016).

Hazelnut (*Corylus* spp.), a common tree that is extensively distributed in Asia, Europe, and North America, has important economic and nutritional value (Özdemir et al., 2001). *Corylus heterophylla* has been widely cultivated in China for centuries. More than four million acres of natural hazel grow in northern China with an annual yield of over 23,000 tons (Hu, 2016a). However, recently, global hazelnut production has declined because of the destructive effects of branch canker (Linaldeddu et al., 2016). Eastern filbert blight, one of the most destructive diseases of *Corylus americana*, was caused by *Anisogramma anomala*, which became commercially important in the 1970s (Gottwald and Cameron, 1980; Pinkerton et al., 1992; Chen et al., 2007). *Cytospora corylicola* has been recognized as a rot agent in European hazelnuts in Italy (Servazzi, 1950; Graniti, 1957; Salerno, 1961; Guerrero and Pérez, 2013a; Guerrero and Pérez, 2013b). Several fungal pathogens have been reported to cause canker and dieback of *Corylus avellana*, including *Anthostoma* (Diatrypaceae), *Diaporthe (Diaporthaceae)*, *Diaporthella* (Gnomoniaceae), *Diplodia*, *Dothiorella* (Botryosphaeriaceae), and *Gnomoniopsis* (Gnomoniaceae) (Guerrero and Pérez, 2013a; Guerrero and Pérez, 2013b; Linaldeddu et al., 2016; Wiman et al., 2019). However, these studies mainly focused on canker and dieback of European hazelnuts, and only the genera *Erysiphe* and *Trichothecium* have been recorded in China (Sun, 2013; Hu, 2016b). *Cytospora coryli* and *Diaporthe coryli*, pathogens of *Corylus mandshurica*, have been collected from Beijing and Shaanxi Province, and are of great significance for research on wild hazelnuts in China (Yang et al., 2020; Zhu et al., 2020). As *Corylus heterophylla* is the main source of hazel products in the Chinese market (Liu et al., 2014a; Liu et al., 2014b), and given the importance of *Corylus* species in the Chinese economy, the fungal pathogens associated with canker and dieback of *C. heterophylla* need to be investigated.

During an investigation of cognitive practices in Beijing, China, 51 strains were isolated from the symptomatic stems and branches of *Corylus heterophylla*. The purpose of this study was to identify these strains using polyphasic approaches and supplement a multi-locus DNA dataset of the pathogens of Chinese wild hazelnut. Additionally, the influences of temperature, pH, and six carbon sources on mycelial growth were determined to evaluate the possible role of these conditions in fungal growth.

## Materials and Methods

### Sampling and Isolation

**Figure 1:**
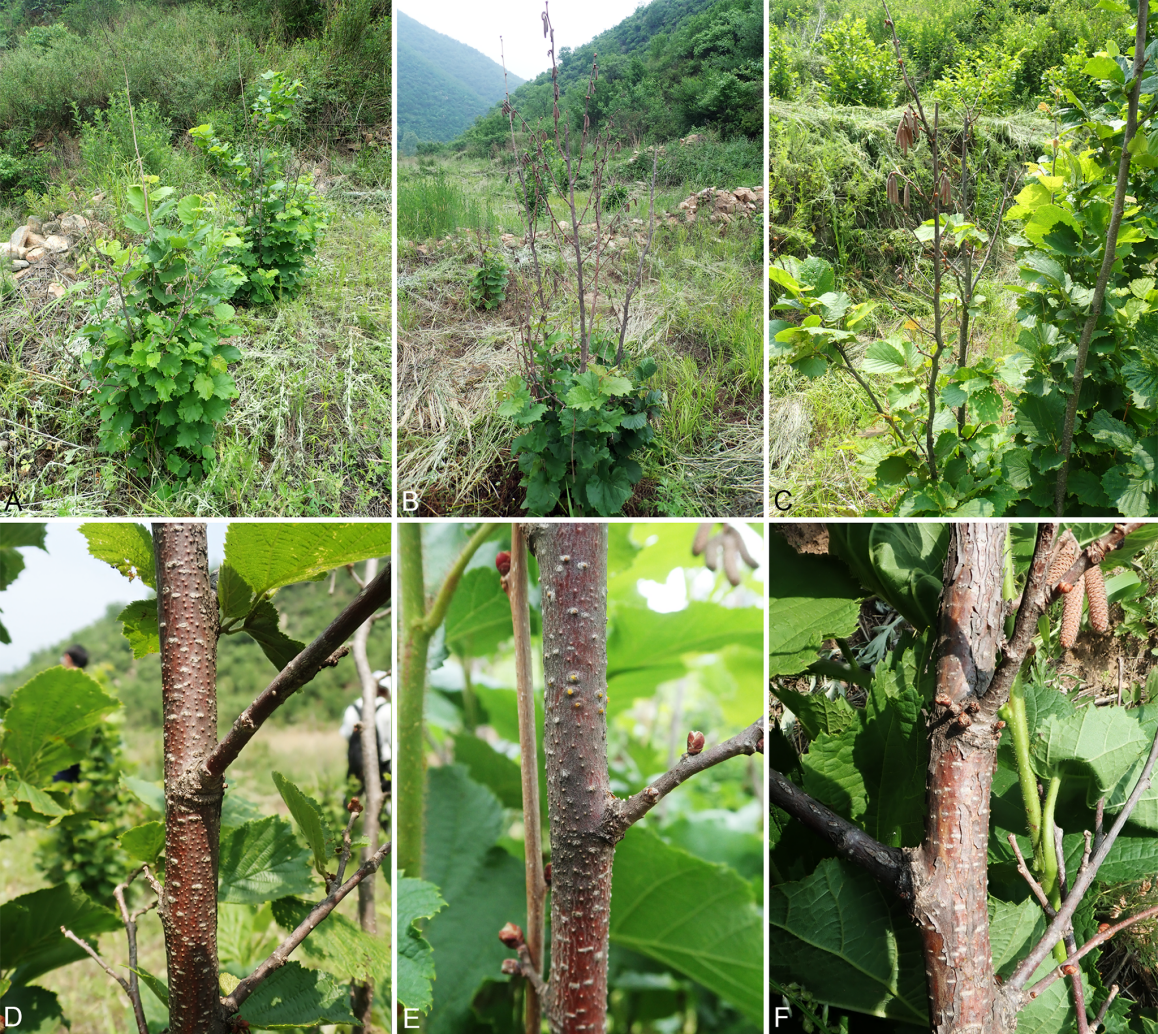
Disease symptoms of *Corylus heterophylla*. **(A–C)** Death of the hazel trees caused by *Cytospora* and *Diaporthe* in the orchards. **(D–F)** Conidiomata on a naturally infected stem in the field.

During the investigation of cognitive practices (June to August, 2019), more than 70 specimens were collected from stems and branches of *Corylus heterophylla* in Huairou District of Beijing, China. These infected stems and branches were collected from three nurseries, expressed typical canker and dieback symptoms with fruiting bodies immersed and erupted through the bark surface when mature (**Figure 1**). Twenty-five specimens were selected and taken to laboratory, observed using a stereo microscope (M205 FA) (Leica microsystem, Wetzlar, Genmany). A total of 51 strains were established by transferring the ascospores or conidial masses from the fruiting bodies on to the surface of PDA (1.8% potato dextrose agar, potato 20 g, dextrose 20 g, agar 17 g, distilled water to complete 1,000 ml) plates with diameter 90 mm. The strains were incubated in darkness at 25°C for 24 h until spores germinated. Single germinating spores were moved to new PDA plates. Specimens have been maintained at the Museum of the Beijing Forestry University (BJFC) and the working Collection of X.L. Fan (CF), housed at the Beijing Forestry University. Living cultures are deposited in the China Forestry Culture Collection Centre (CFCC).

### Morphological Observations

Species identification was based on morphological features of the fruiting bodies, which was produced on the infected plant tissues, including stromata (arrangement and size), conceptacle (presence or absence), conidiomata (color, size, and shape), ostioles (number and diameter), locules (number and arrangement type), conidiophores, and conidia (size and shape), supplemented by cultural characteristics such as colony color, texture, and the presence or absence of airborne hyphae (Mostert et al., 2001; Zhang et al., 2007; Zhu et al., 2020). The morphological features were observed under a Leica stereomicroscope (M205 FA) (Leica microsystem, Wetzlar, Genmany). Micro-morphological observations determined by a Nikon Eclipse 80i compound microscope. Measuring 30 conidiomata/ascomata and 50 conidia/ascospores, determined by length, width, and length/width ratio (L/W ratio). Recording the colony diameters and describing the color was based on the color charts of Rayner (1970) after 1–2 weeks on PDA in darkness. The results were edited manually by Adobe Bridge CS v. 6 and Adobe Photoshop CS v. 5.

### DNA Extraction and PCR Amplification

Scrapping the mycelium from the cellophane for DNA extraction after three days dark-incubation at 25°C. Using the modified CTAB method to extract genomic DNA (Doyle and Doyle, 1990). Using a 20 μl system of 10 μl Mix (Promega), 7 μl ddH_2_O, 1 μl upstream primer, 1 μl downstream primer, and 1 μl template DNA to conduct polymerase chain reaction (PCR) in order to amplify gene fragments. The primers and PCR conditions are set in **Table S1**. Electrophoretic separation was conducted for the PCR amplification products in 2% agarose gels with a DNA maker 2,000 bp (Takara Biotech). Using an ABI PRISM^®^ 3730XL DNA Analyzer with BigDye^®^ Terminater Kit v. 3.1 (Invitrogen) at the Shanghai Invitrogen Biological Technology Company (Beijing, China) to conduct the DNA sequences. In order to acquire a consensus sequence of sequences obtained from forward and reverse primer pairs, Seqman v. 9.0.4 (DNASTAR Inc., Madison, WI, United States) was used.

### Phylogenetic Analyses

Using MAFFT v. 6 (Katoh and Standley, 2013) to align ITS sequence data and editing it manually with MEGA v. 6.0 (Tamura et al., 2013), the current strains were preliminarily identified as *Cytospora* and *Diaporthe* species. To clarify their further phylogenetic position, five genes (ITS, *act*, *rpb2*, *tef1-α*, and *tub2* for *Cytospora*; ITS, *cal*, *his3*, *tef1-α*, and *tub2* for *Diaporthe*) for each genus were combined and aligned to compare with other strains in GenBank secondly. Generating subsequent alignments for each gene and adjusting them manually. Excluding the ambiguously aligned sequences from analyses. Reference sequences were retrieved from recent publications (Fan et al., 2014a; Fan et al., 2014b; Fan et al., 2015a; Fan et al., 2015b; Lawrence et al., 2017; Fan et al., 2020; Pan et al., 2020; Zhu et al., 2020; Pan et al., 2021). *Diaporthe vaccinii* (CBS 160.32) was included as the outgroup in *Cytospora* analysis (**Table S2**) and *Diaporthella corylina* (CBS 121124) was included in *Diaporthe* analysis (**Table S3**). All the datasets were performed using PAUP v. 4.0b10 for the maximum parsimony (MP) method (Swofford, 2003), RAxML for the maximum likelihood (ML) method (Stamatakis, 2006), and MrBayes v. 3.1.2 for the Bayesian Inference (BI) method (Ronquist and Huelsenbeck, 2003) for the phylogenetic analyses. All novel sequences derived from this study were deposited in MycoBank (www.mycobank.org) (Crous et al., 2004). All sequences from this study were submitted in GenBank, as shown in **Tables S2** and **S3**. The multi-gene sequence alignment files were deposited in TreeBASE (www.treebase.org; accession number: S276989).

### Testing the Influence of Temperature, pH, and Carbon on Mycelium Growth

All 51 strains we collected were identified as three species of *Cytospora* and two of *Diaporthe* through morphological and phylogenetic analyses. We selected representative strains of each species to assess the influences of temperature, pH, and carbon sources on mycelium growth incubated in the dark. A mycelial plug with 5-mm-diameter was transferred on to the center of a 90 mm PDA plate and incubated in an environment of 0–40°C with a 5°C gradient (i.e., 0, 5, 10, 15, 20, 25, 30, 35, and 40°C), three repeats were set for each treatment (Fang, 1979; Zhou et al., 2020). The pH values of the PDA were regulated to a range of 2.0–12.0 using 1 mol/L NaOH and 1 mol/L HCl, to obtain pH 2.0, 3.0, 4.0, 5.0, 6.0, 7.0, 8.0, 9.0, 10.0, 11.0, and 12.0. Taking a mycelial plug to PDA plate with the same methods as temperature tests and incubating in darkness at 25°C, three replicates were also set. The strains of the five species were incubated in darkness at 25°C on PDA (the 20 g dextrose were replaced by 20 g fructose, galactose, maltose, sucrose, or xylose) plates to assess the utilization of these compounds as carbon sources (Zhao et al., 2019; Zhou et al., 2020). The colony diameter was measured in millimeters every 24 h for four days of incubation, and converting the data to radial growth to assess the effects of temperature, pH, and carbon sources on mycelial growth (Zhao et al., 2019). All data were analyzed with one-way ANOVA and Post Hoc of LSD and Tukey using IBM SPSS Statistics v. 22.0 (IBM Inc., Armonk, NY, USA). A *p*-value < 0.05 was considered significant. Graph with SE-bar were conducted in order to explain the difference in mycelium growth under different conditions.

## Results

### Isolation

During the investigation of cognitive practices, we isolated a total of 51 strains from infected stems and branches of hazelnut trees (**Table S4**). Among the 51 strains, 14 strains were identified as *Cytospora* species (37%) and 37 strains were *Diaporthe* (73%). *Diaporthe corylicola* is the main species observed on *Corylus heterophylla* (64.71%), followed by *Cytospora curvispora* (11.76%). The rest were identified as *Cytospora corylina*, *Cytospora leucostoma*, and *Diaporthe eres*, four strains of each species, accounting 7.84% of total, respectively.

### Phylogeny

**Figure 2:**
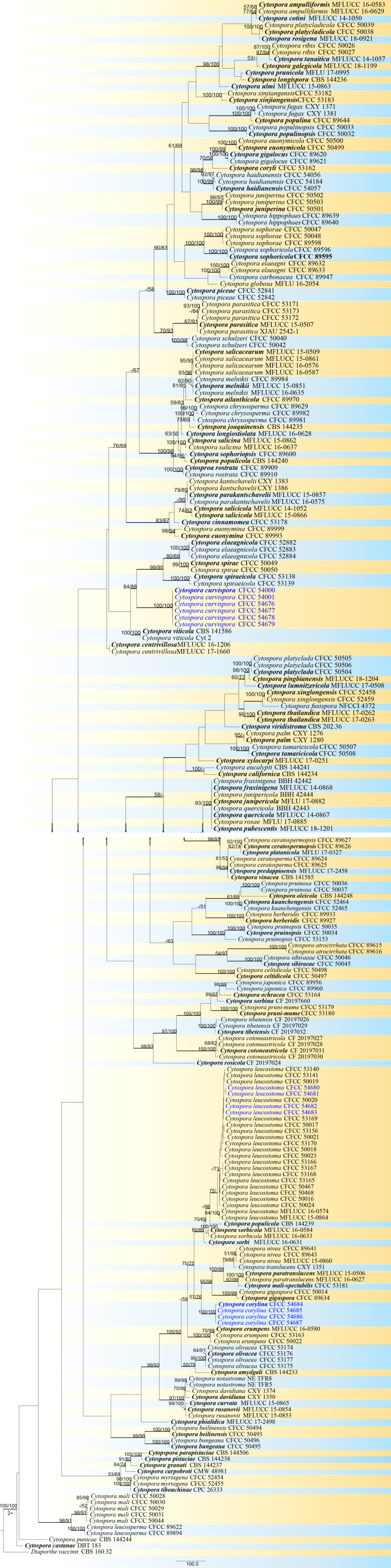
Phylogram of *Cytospora* based on combined five genes (ITS, *act*, *rpb2*, *tef1-α*, and *tub2*). MP/ML-BS ≥50% are shown at first/second positions. Branches with BPP ≥0.95 are thickened. Strains in this study are in blue. Ex-type strains are in bold.

Totally 14 strains of *Cytospora* obtained from hazelnut and other 224 strains retrieved from recent publications were used in the phylogenetic analyses (Spielman, 1985; Adams and Taylor, 1993; Adams et al., 2002; Adams et al., 2005; Lawrence et al., 2017; Fan et al., 2018; Fan et al., 2020; Jiang et al., 2020; Pan et al., 2020; Zhou et al., 2020; Zhu et al., 2020; Pan et al., 2021). The sequence datasets for the five genes (ITS, *act*, *rpb2*, *tef1-α*, and *tub2*) were performed in individual and combined analyses. The single gene region analysis is very similar to the tree topologies of the combined analyses. The phylogram generated here indicated 237 ingroup taxa including 3,686 characters (622 for ITS, 457 for *act*, 1,076 for *rpb2*, 775 for *tef1-α*, and 756 for *tub2*) in the multi-locus analyses, of which 2,010 characters were constant, 191 variable characters were parsimony uninformative and 1,485 characters were parsimony informative. MP analysis generated 200 parsimonious trees (TL = 9,878, CI = 0.307, RI = 0.812, RC = 0.249) and the first one is selected and shown in **Figure 2**. All trees of ML and BI analyses were in agreement and no significant difference with MP tree. MP/ML-BS (MP/ML bootstrap support) ≥50% were shown, and branches with BPP (BI posterior probability) ≥0.95 were thickened in the phylogram.

**Figure 3:**
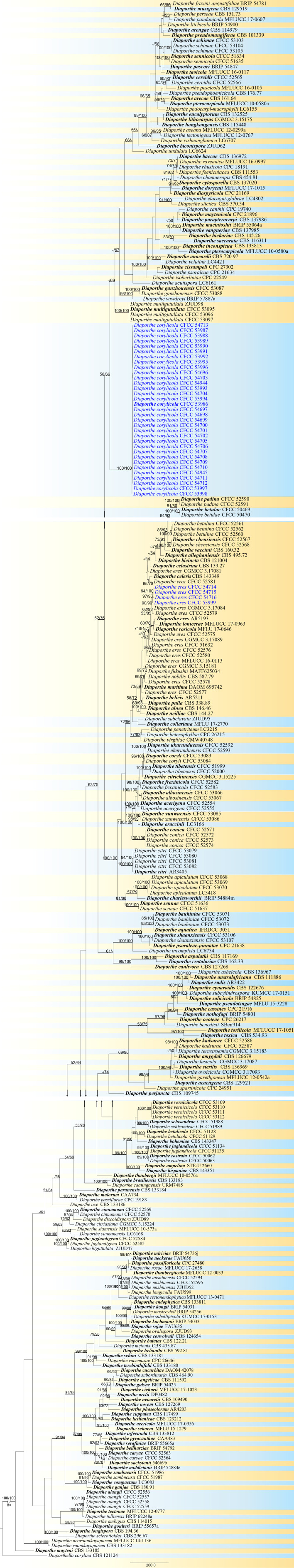
Phylogram of *Diaporthe* based on combined five genes (ITS, *cal*, *his3*, *tef1-α*, and *tub2*). MP/ML-BS ≥50% are shown at first/second positions. Branches with BPP ≥0.95 are thickened. Strains in this study are in blue. Ex-type strains are in bold.

Thirty-seven strains of *Diaporthe* from hazelnut were aligned with 253 strains supplement from recent publications. The five genes (ITS, *cal*, *his3*, *tef1-α*, and *tub2*) in individual and combined were used to clarify the phylogenetic position of these *Diaporthe* species. The tree topologies generated by the MP, ML, and BI analyses were similar. All strains counted 2,983 characters including gaps (613 for ITS, 587 for *cal*, 578 for *his3*, 645 for *tef1-α*, and 560 for *tub2*), of which 1,233 characters are constant, 360 variable characters are parsimony uninformative 1,390 characters are parsimony informative. MP analysis generated 100 parsimonious trees (TL = 1,826, CI = 0.267, RI = 0.788, RC = 0.210), the first one is selected and presented in **Figure 3**. The support values were shown and branches were thickened in the phylogram at the same standard as **Figure 2**.

The current 14 strains of *Cytospora* clustered in three clades representing three species, of which one was known as *C. leucostoma* and two were distinguished from all other known taxa with high support values (MP/ML/BI = 100/100/1) as two clades. Thus, we described the two clades as *C. corylina*, *C. curvispora* here. Four strains were assigned to *Diaporthe eres*, and the remaining 33 were classified as one new species, *D. corylicola*, representing a monophyletic clade with high support values (MP/ML/BI = 100/100/1).

### Taxonomy

**Figure 4:**
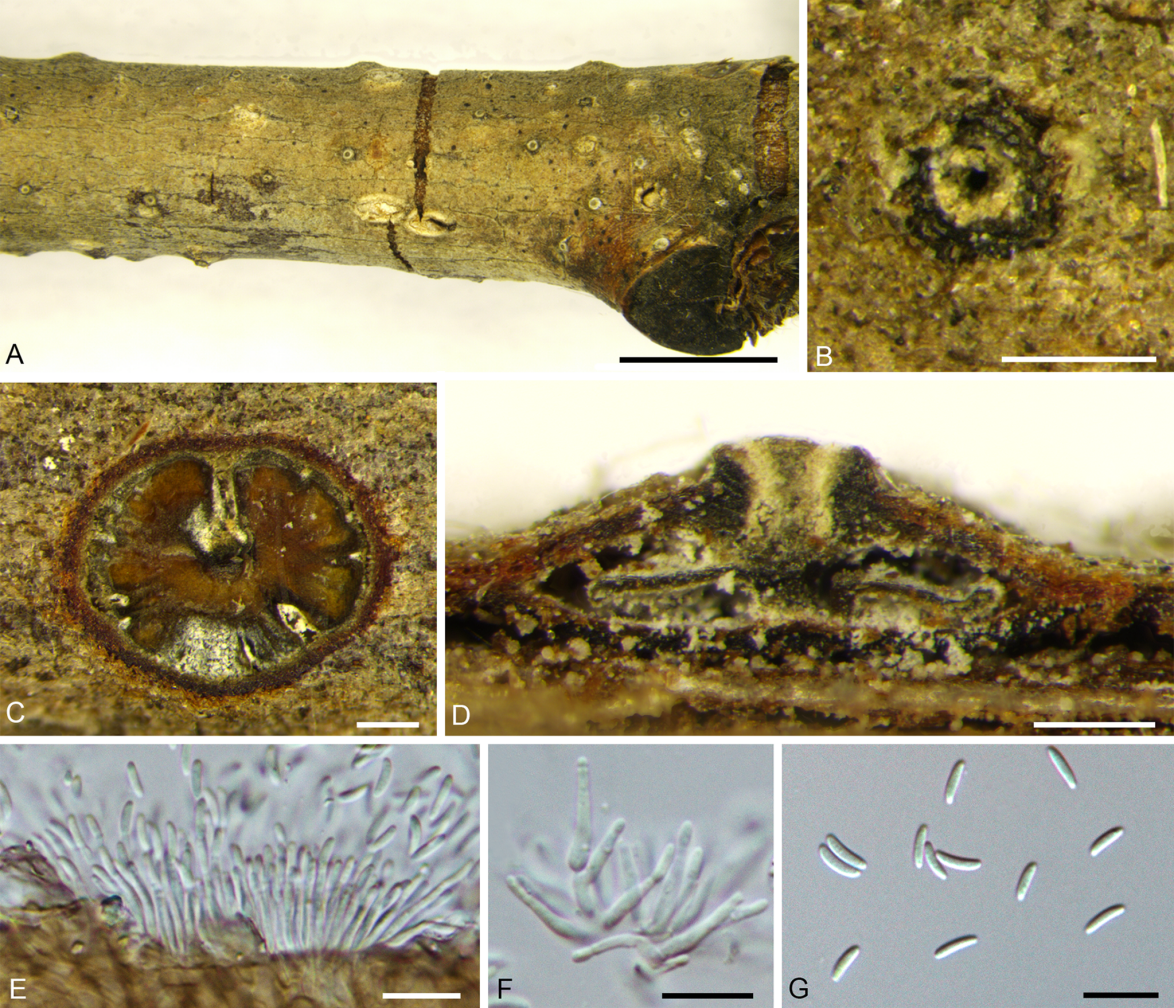
*Cytospora corylina* from *Corylus heterophylla*. **(A, B)** Habit of conidiomata on twig. **(C)** Transverse section of conidioma. **(D)** Longitudinal section through conidioma. **(E, F)** Conidiophores and conidiogenous cells. **(G)** Conidia. Scale bars: 3 mm **(A)**; 200 µm **(B–D)**; 10 µm **(E–G)**.

***Cytospora corylina*** H. Gao & X.L. Fan, sp. nov. **Figure 4**.

MycoBank MB 838643.

*Holotype*: CHINA, Beijing City, Huairou District, Forestry Center of Beijing University of Agriculture, 116°26′16.66″E, 40°52′52.18″N, from branches of *Corylus heterophylla*, June, 2019, *H. Gao* & *X.L. Fan* (**holotype** BJFC CF20210114), ex-type living culture CFCC 54684.

*Etymology*: Named after the host genus on which it was collected, *Corylus*.

*Description*: Necrotic tissues on branches of *Corylus heterophylla*. Sexual morph: not observed. Asexual morph: Stromata immersed in bark. Pycnidial stromata ostiolated, scattered, erupted slightly through the bark surface. Multiple locules, conceptacle absent. 850–1,280 (av. = 913, n = 30) µm in diameter. Ectostromatic disc gray with one ostiole in center, discoid, circular to ovoid, 180–270 (av. = 214, n = 30) µm in diameter. Ostiole slight-brown, inconspicuous, at the same level as the disc surface. 110–185 (av. = 135, n = 30) µm in diameter. Locules numerous, circular to irregular with common walls generally invaginated. Conidiophores branched, occasionally unbranched, hyaline, approximately cylindrical, 8.0–13.5 × 1.0–2.5 (av. = 10.8 ± 1.6 × 1.6 ± 0.4, n = 30) µm, sometimes reduced to conidiogenous cells. Conidiogenous cells enteroblastic, phialidic, subcylindrical to cylindrical. Conidia hyaline, allantoid, smooth, aseptate, thin-walled, 3.5–7.5 × 1.0–1.5 (av. = 5.8 ± 0.8 × 1.3 ± 0.2, n = 50) µm, L/W ratio 3.61–4.52 (av. = 4.27 ± 0.17).

*Culture characteristics*: Colonies are initially white, reaching 90 mm after two days, slightly fawn in center and becoming nearly fucous black after 30 days. Colonies concentric circles, with a thick texture, and aerial mycelium lacked. Conidiomata distributed on PDA surface irregularly surrounded by dark mycelium (**Figure 9A**).

*Other specimens examined*: CHINA, Beijing City, Huairou District, Forestry Center of Beijing University of Agriculture, 116°26′21.62″E, 40°52′47.58″N, from branches of *Corylus heterophylla*, June, 2019, *H. Gao* & *X.L. Fan* (BJFC C20210115), living culture CFCC 54685 and CFCC 54686; *ibid.* BJFC CF20210116, living culture CFCC 54687.

*Notes*: *Cytospora corylina* was collected from *Corylus heterophylla* in Beijing, China. It is significantly different from *Cytospora coryli* isolated by Zhu et al. (2020) in morphology. *Cytospora coryli* has a flat conidiomata and inconspicuous ostiole. In terms of culture morphology, The colonies of *C. coryli* are brown, conidiomata distributed radially on colony surface. The four strains representing *Cytospora corylina* clustered as a single lineage and appeared to be the most closely related to *C. gigaspora*, *C. mali-spectabilis*, *C. nivea*, *C. paratranslucens*, and *C. translucens*, with support values of MP/ML/BI = 57/76/0.99. However, this strain could be distinguished from the five most related species by the conidiomata (*C. gigaspora* with a flat locule, *C. mali-spectabilis* with a column lenticular tissue in the center, and *C. nivea* with a dark conceptacle) (Fan et al., 2015b; Pan et al., 2020). In addition, *C. corylina* has a smaller conidia size (3.5–7.5 × 1–1.7 µm) than *C. gigaspora* (8.9–12.1 × 1.9–2.9 µm), *C. mali-spectabilis* (9.0–10.0 × 1.5–2 µm), *C. nivea* (6.2–9.2 × 1.7–2.4 µm), and *C. paratranslucens* (6.5–7.3 × 1.3–1.5 µm) (Adams et al., 2006; Fan et al., 2015b; Norphanphoun et al., 2017; Pan et al., 2020). Therefore, we describe this species as novel based on DNA sequence data and morphology.

**Figure 5:**
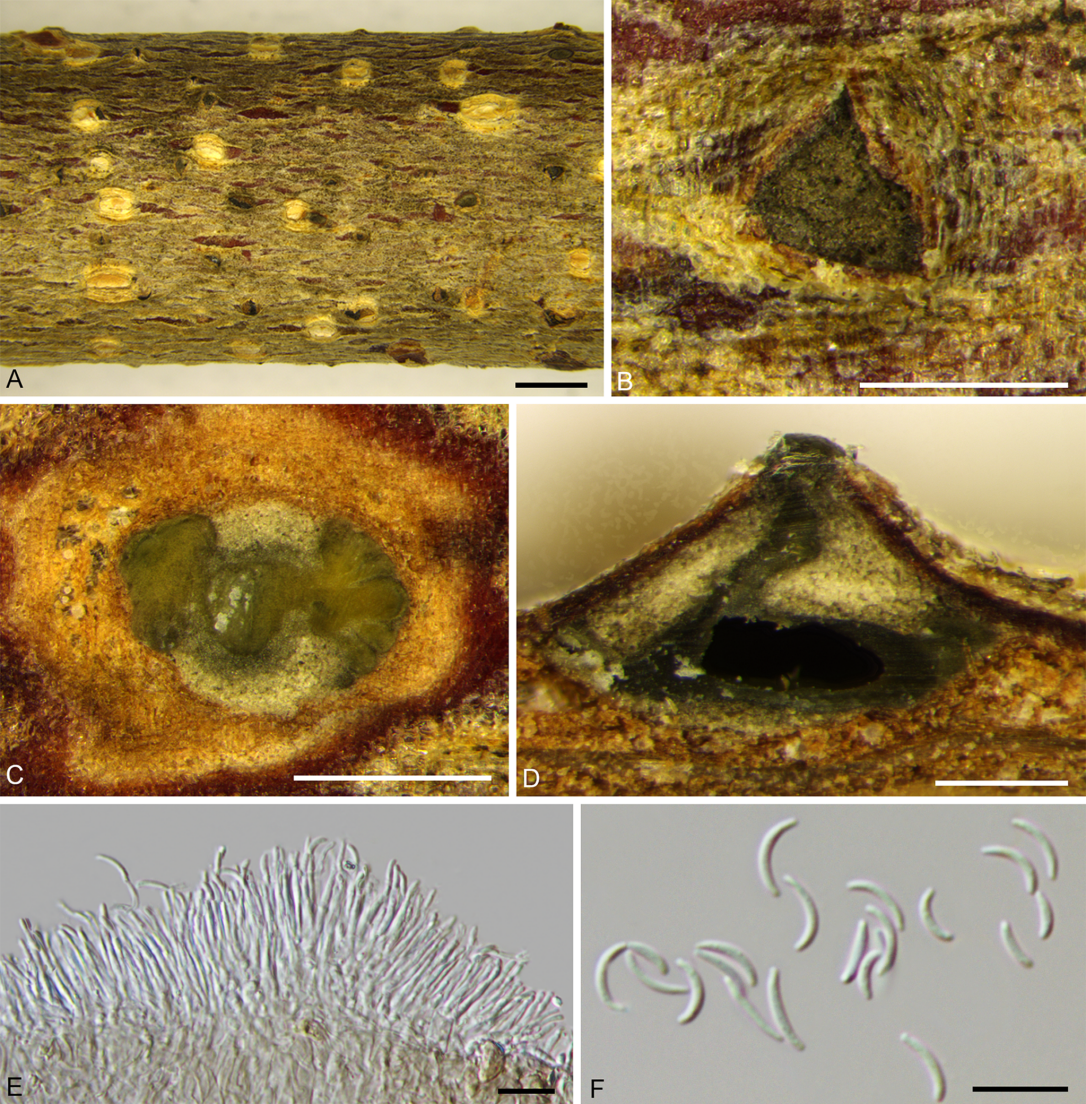
*Cytospora curvispora* from *Corylus heterophylla*. **(A, B)** Habit of conidiomata on twig. **(C)** Transverse section of conidioma. **(D)** Longitudinal section through conidioma. **(E)** Conidiophores and conidiogenous cells. **(F)** Conidia. Scale bars: 2 mm **(A)**; 500 µm **(B–D)**; 10 µm **(E, F)**.

***Cytospora curvispora*** H. Gao & X.L. Fan, sp. nov. **Figure 5**.

MycoBank MB 838641.

*Holotype*: CHINA, Beijing City, Huairou District, Forestry Center of Beijing University of Agriculture, 116°26'23.64"E, 40°52'48.37"N, from branches of *Corylus heterophylla*, June, 2019, *H. Gao* & *X.L. Fan* (**holotype** BJFC CF20210110), ex-type living culture CFCC 54000.

*Etymology*: Named after the character of its curved conidia.

*Description*: Necrotic tissues on stems and branches of *Corylus heterophylla*. Sexual morph: not observed. Asexual morph: Stromata immersed in bark. Conidiomata pycnidial, scattered, conical, erupted through the bark surface when mature, locules multiple. Conceptacle absent, diameter 1,080–1,700 (av. = 1,423, n = 30) µm. Ectostromatic disc black-brown, discoid, circular to ovoid, 480–660 (av. = 526, n = 30) µm in diameter, with one ostiole. Ostiole dark-gray to black, inconspicuous, slightly curved, at the same level as the disc surface, 40–70 (av. = 58, n = 30) µm in diameter. Locules numerous, circular to irregular with common wall generally invaginated. Conidiophores unbranched, barely branched, hyaline, approximately cylindrical, 9.5–14.0 × 1.0–1.5 (av. = 12.5 ± 1.3 × 1.1 ± 0.3, n = 30) µm, sometimes reduced to conidiogenous cells. Conidiogenous cells enteroblastic, phialidic, subcylindrical to cylindrical. Conidia hyaline, elongate­allantoid to falcate, smooth, aseptate, thin-walled, 4.5–8.5 × 1.0–1.5 (av. = 6.7 ± 1.0 × 1.3 ± 0.2, n = 50) µm, L/W ratio 4.88–6.04 (av. = 5.19 ± 0.22).

**Figure 9:**
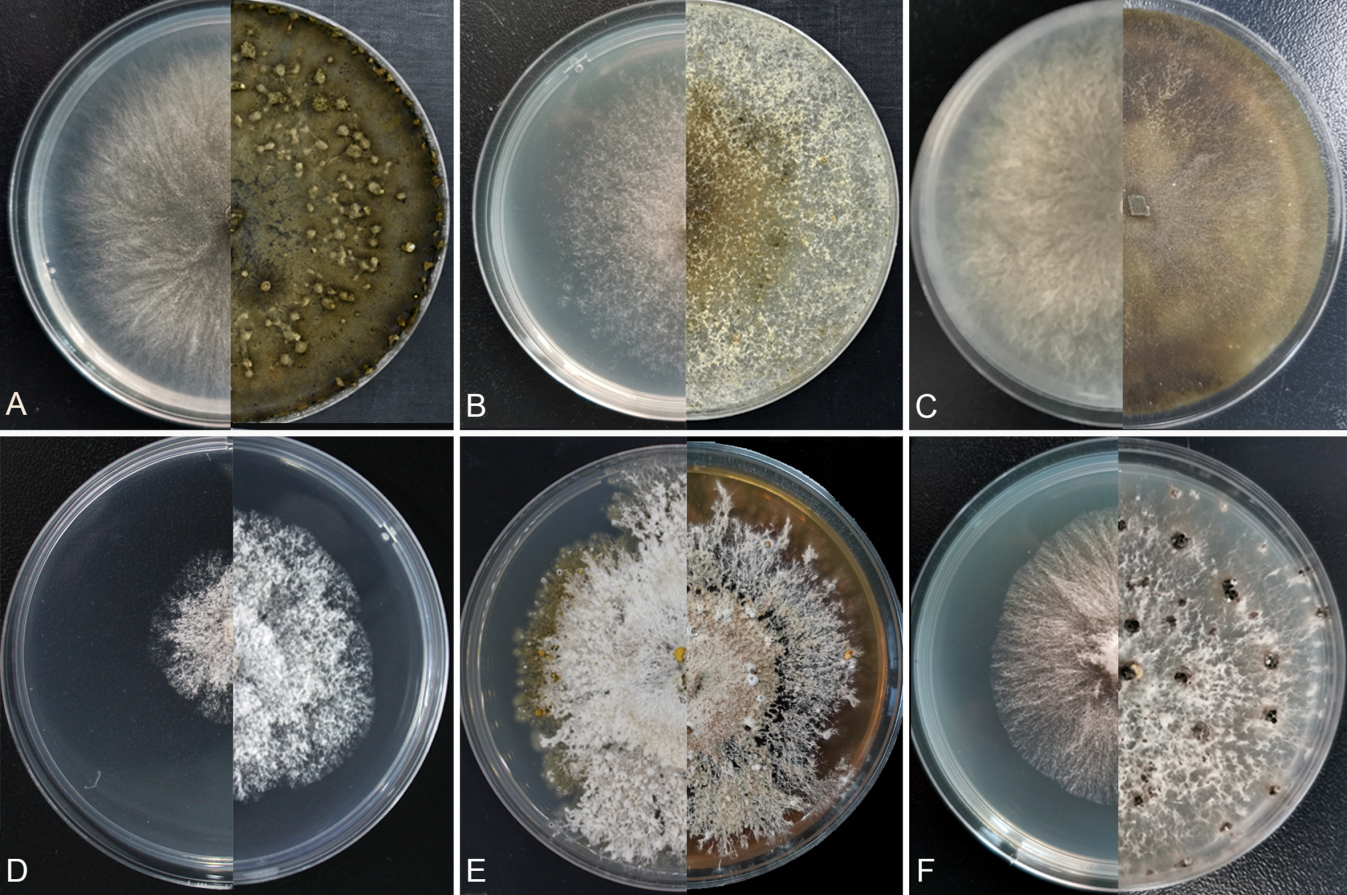
Cultures on potato dextrose agar (PDA). **(A)** *Cytospora corylina*. **(B)** *Cytospora curvispora*. **(C)** *Cytospora leucostoma*. **(D, E)** *Diaporthe corylicola*. **(F)** *Diaporthe eres*. Days of incubation: three days at left and 30 days at right **(A–C)**, **(F)**; three days at left and seven days at right **(D)**; 15 days at left and 30 days at right **(E)**.

*Culture characteristics*: Cultures are initially white, thin, reaching 70 mm after three days, turning slightly honey after 30 days and deepened continuously. Colonies are uniform, aerial mycelium lacked. Conidiomata distributed irregularly on PDA surface (**Figure 9B**).

*Other specimens examined*: CHINA, Beijing City, Huairou District, Forestry Center of Beijing University of Agriculture, 116°26'28.24"E, 40°52'48.73"N, from branches of *Corylus heterophylla*, June, 2019, *H. Gao* & *X.L. Fan* (BJFC CF20210111), living culture CFCC 54001; CHINA, Beijing City, Huairou District, Forestry Center of Beijing University of Agriculture, 116°26'22.83"E, 40°52'50.57"N, from branches of *C. heterophylla*, June, 2019, *H. Gao* & *X.L. Fan* (BJFC CF20210118), living culture CFCC 54676 and CFCC 54677; CHINA, Beijing City, Huairou District, Forestry Center of Beijing University of Agriculture, 116°26'20.22"E, 40°52'41.15"N, from stems of *C. heterophylla*, June, 2019, *H. Gao* & *X.L. Fan* (BJFC CF20210113), living culture CFCC 54678 and CFCC 54679.

*Notes*: *Cytospora curvispora* was isolated from *Corylus heterophylla* in Beijing, China. We can distinguish it from *C. coryli* by its curved conidia and conical conidiomata. In the phylogeny analysis, *Cytospora curvispora* clustered with *C. elaeagnicola*, *C. spiraeae*, and *C. spiraeicola* with support values of MP/ML/BI = 84/88/0.99. However, differences in their distribution and morphology were identified. *Cytospora spiraeae* and *C. spiraeicola* were isolated from *Spiraea salicifolia*, collected from Gansu Province and Beijing, respectively. *Cytospora elaeagnicola* was collected from branches of *Elaeagnus angustifolia* in the Xinjiang Uygur Autonomous Region. Morphologically, the conidia of *C. curvispora* are more curved and with a smaller width (1.0–1.5 µm for *C. curvispora*, 1.5–2.0 µm for *C. elaeagnicola*, 2.0–2.5 µm for *C. spiraeae*, 2.5–3.5 µm for *C. spiraeicola*). In addition, *C. elaeagnicola* colonies are white and have a thick texture at the center, becoming thinner at the edges; *C. spiraeae* is fawn and felt-like and *C. spiraeicola* is buff to hazel with a heterogeneous texture (Zhu et al., 2018; Zhang et al., 2019; Zhu et al., 2020). Thus, we described this finding as novel based on morphological and phylogenetic analyses.

**Figure 6:**
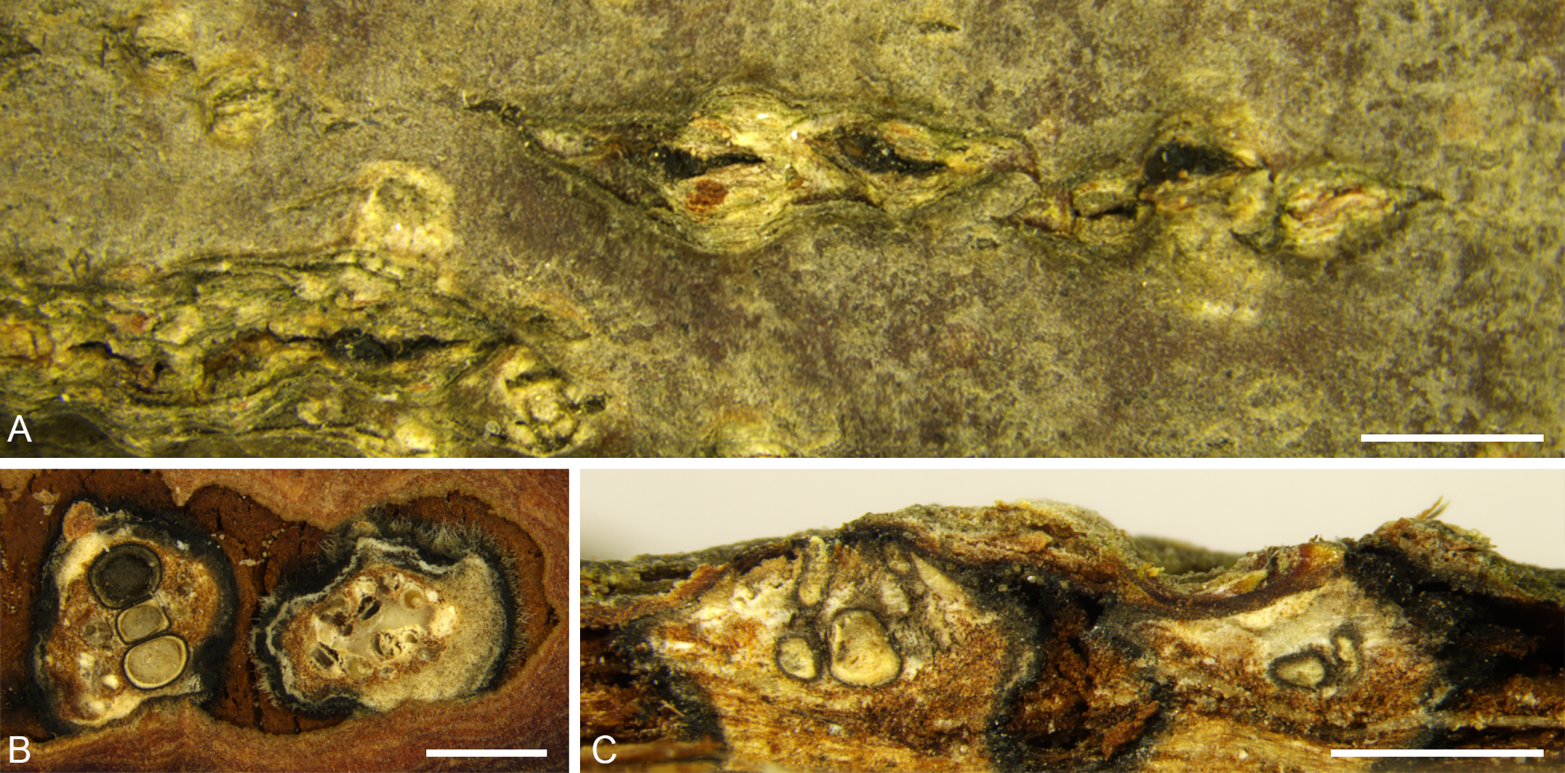
*Cytospora leucostoma* from *Corylus heterophylla*. **(A)** Habit of ascomata on twig. **(B)** Transverse section of ascomata. **(C)** Longitudinal section through ascomata. Scale bars: 2 mm **(A)**; 1 mm **(B, C)**.

***Cytospora leucostoma*** (Pers.) Sacc., Michelia, 2: 264, 1881. **Figure 6**.

*Description*: see Fan et al. (2020).

*Culture characteristics*: Colonies are white initially and change to dark green to dark after seven days. Growing up to 80 mm after three days. Colonies have a uniform texture, thick, aerial mycelium lacked (**Figure 9C**).

*Specimens examined*: CHINA, Beijing City, Huairou District, Forestry Center of Beijing University of Agriculture, 116°26′15.67″E, 40°52′40.18″N, from branches of *Corylus heterophylla*, June, 2019, *H. Gao* & *X.L. Fan* (BJFC CF20210117), living culture CFCC 54680 and CFCC 54681; CHINA, Beijing City, Huairou District, Forestry Center of Beijing University of Agriculture, 116°26′36.65″E, 40°52′32.14″N, from branches of *C. heterophylla*, June, 2019, *H. Gao* & *X.L. Fan* (BJFC CF2021118), living culture CFCC 54682 and CFCC 54683.

*Notes*: *Cytospora leucostoma* commonly causes branch canker in Rosaceae in China (Fan et al., 2020; Pan et al., 2020). In the current study, two specimens were collected from the infected branches of the Chinese hazelnut, and fruiting bodies were observed. However, the specimens were not fresh, and little information is available on the micromorphology. Thus, we identified the strains as *C. leucostoma*, based on ascostromata with conceptacle, culture characteristics, and DNA data.

**Figure 7:**
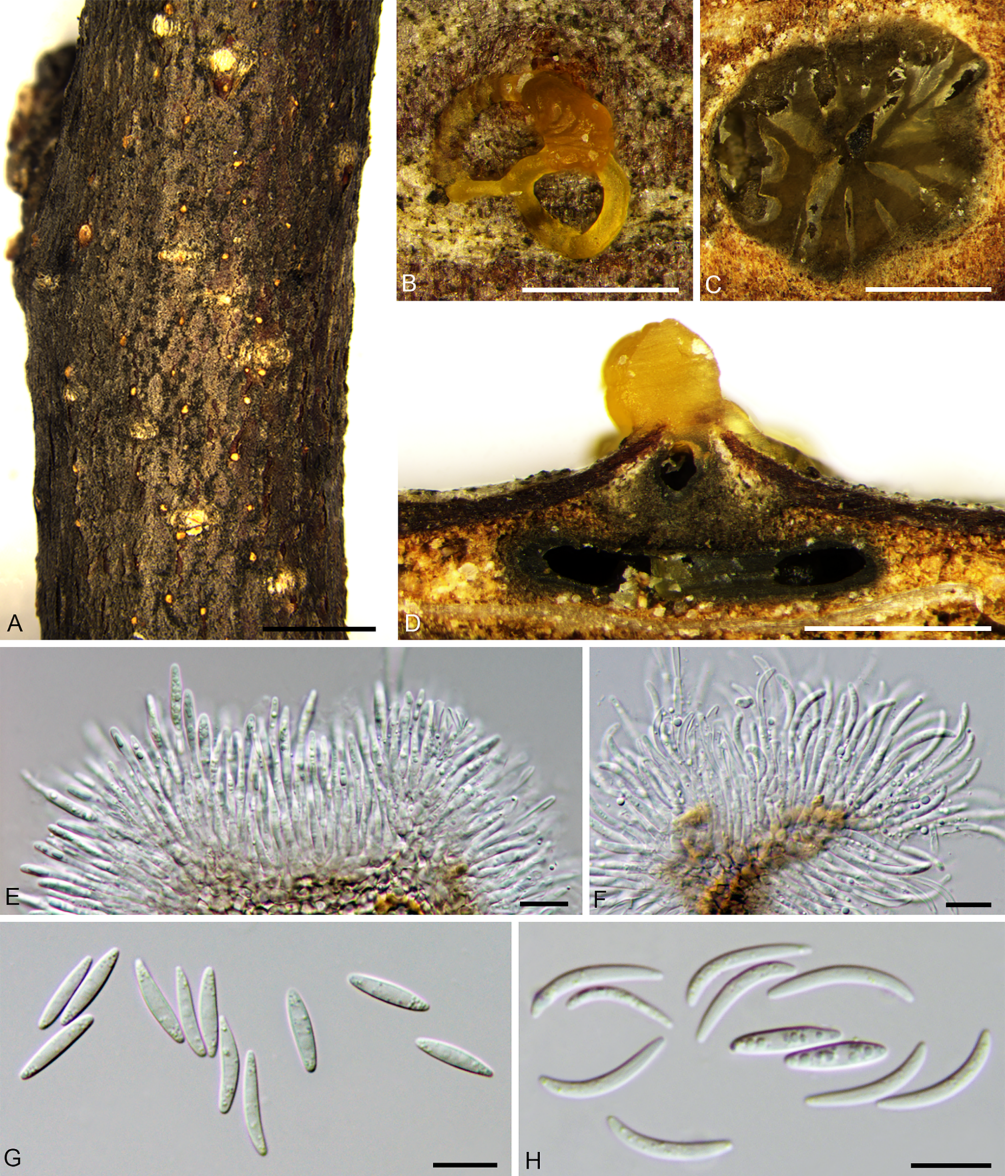
*Diaporthe corylicola* from *Corylus heterophylla*. **(A, B)** Habit of conidiomata on twig. **(C)** Transverse section of conidiomata. **(D)** Longitudinal section through conidioma. **(E, F)** Conidiogenous cells and conidia. **(G, H)** Alpha and gamma conidia. Scale bars: 3 mm **(A)**; 500 µm **(B–D)**; 10 µm **(E–H)**.

***Diaporthe corylicola*** H. Gao & X.L. Fan, sp. nov. **Figure 7**.

MycoBank MB 838644.

*Holotype*: CHINA, Beijing City, Huairou District, Forestry Center of Beijing University of Agriculture, 116°26′18.55″E, 40°52′38.27″N, from stems of *Corylus heterophylla*, June, 2019, *H. Gao* & *X.L. Fan* (**holotype** BJFC CF20210121), ex-type living culture CFCC 53986.

*Etymology*: Named after the host genus on which it was collected, *Corylus*.

*Description*: Necrotic tissues on stems and branches of *Corylus heterophylla*. Asexual morph: Stromata immersed in bark. Pycnidial stromata ostiolated, scattered, discoid to conical, erumpent slightly through the surface of bark at maturity, with single locule. Conceptacle absent, 750–1,300 (av. = 991, n = 30) µm in diameter. Ectostromatic disc buff or orange with only one ostiole in center, covered by orange discharged conidial masses, discoid, circular to ovoid, 175–270 (av. = 206, n = 30) µm in diameter. Ostiole dark-brown, conspicuous, at the same level as the disc surface, 130–150 (av. = 141, n = 30) µm in diameter. Conidiogenous cells enteroblastic, phialidic, subcylindrical to cylindrical, 16.0–24.0 × 1.5–2.5 (av. = 19.4 ± 2.5 × 2.0 ± 0.2, n = 30) µm. Alpha conidia are aseptate, hyaline, fusiform, multi-guttulate, rarely 2 guttulate and smooth, 11.0–16.5 × 2.0–3.5 (av. = 13.8 ± 1.3 × 2.8 ± 0.3, n = 50) µm, L/W ratio 4.58–5.41 (av. = 4.87 ± 0.19). Gamma conidia hyaline, multi-guttulate, subcylindrical with a nearly rounded apex, 13.0–19.5 × 1.5–2.5 (15.3 ± 1.1 × 1.7 ± 0.2, n = 50) µm, L/W ratio 6.68–12.61 (av. = 8.92 ± 1.28). Beta conidia undetermined.

*Culture characteristics*: Colonies are white initially, only 25 mm after three days and going to buff after 15 days. Colonies are felty with thick texture, aerial mycelium lacked and conidiomata are randomly distributed fat the marginal area, with orange conidial drops oozing out of the ostioles (**Figures 9D, E****)**.

*Other specimens examined*: CHINA, Beijing City, Huairou District, Forestry Center of Beijing University of Agriculture, 116°26′13.81″E, 40°52′58.11″N, from stems of *Corylus heterophylla*, June, 2019, *H. Gao* & *X.L. Fan* (BJFC CF20210122), living culture CFCC 53987; *ibid.* BJFC CF20210123, living culture CFCC 53988 to 53990; BJFC CF20210134, living culture CFCC 54944 and CFCC 54945; CHINA, Beijing City, Huairou District, Forestry Center of Beijing University of Agriculture, 116°26′25.25″E, 40°52′29.83″N, from stems of *C. heterophylla*, June, 2019, *H. Gao* & *X.L. Fan* (BJFC CF20210124), living culture CFCC 53991 to CFCC 53993; *ibid.* BJFC CF20210125, living culture CFCC 53994 and CFCC 54704; BJFC CF20210126, living culture CFCC 53995; CHINA, Beijing City, Huairou District, Forestry Center of Beijing University of Agriculture, 116°26′21.21″E, 40°52′38.02″N, from branches of *C. heterophylla*, June, 2019, *H. Gao* & *X.L. Fan* (BJFC CF20210127), living culture CFCC 53996 to 53998; *ibid.* BJFC CF20210128, living culture CFCC 554696 and CFCC 54697; BJFC CF20210129, living culture CFCC 54698 to CFCC 54701; BJFC CF20210130, living culture CFCC 54702 to CFCC 54705; CHINA, Beijing City, Huairou District, Forestry Center of Beijing University of Agriculture, 116°26′28.67″E, 40°52′36.72″N, from stems of *C. heterophylla*, June, 2019, *H. Gao* & *X.L. Fan* (BJFC CF20210131), living culture CFCC 54706 to CFCC 54708; *ibid.* BJFC CF20210132, living culture CFCC 54709 to CFCC 54712; BJFC CF20210133, living culture CFCC 54713.

*Notes*: *Diaporthe corylicola* was isolated from *Corylus heterophylla* in Beijing, China. The cluster represented a single lineage with high support (MP/ML/BI = 100/100/1). The culture morphology was similar to that of *D. coryli*, which was isolated from *Corylus mandshurica*, but alpha conidia were longer and thinner (11.0–16.5 × 2.0–3.5 *vs*. 11.5–13 × 3–3.5 µm) (Yang et al., 2020). Phylogenetically, there was high contrast between *Diaporthe coryli* and *D. corylicola*, with 36/610 for ITS, 77/584 for *cal*, 63/575 for *his3*, 61/642 for *tef1-α*, and 61/556 for *tub2*. Thus, we describe this species as novel.

**Figure 8:**
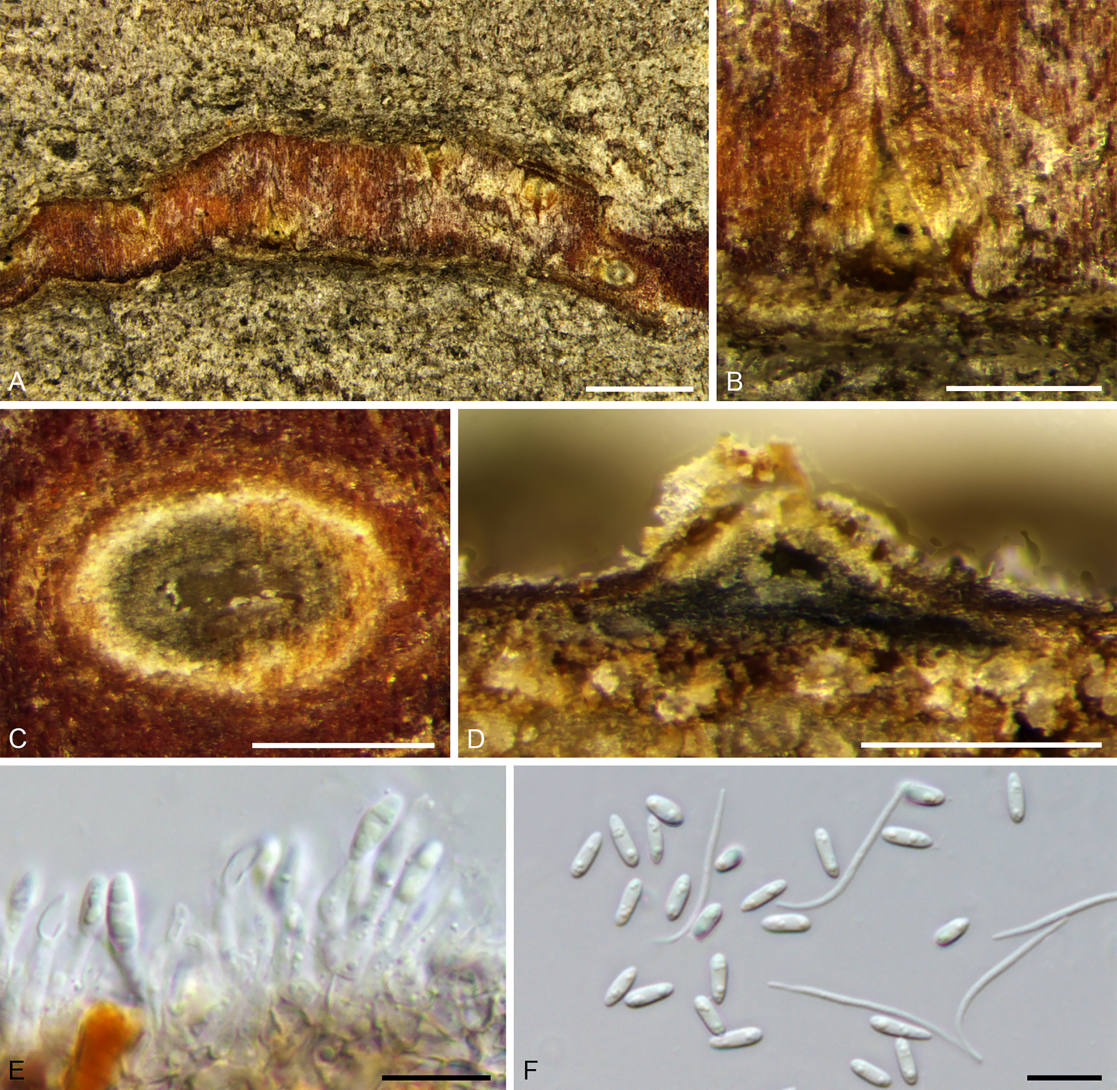
*Diaprthe eres* from *Corylus heterophylla*. **(A, B)** Habit of conidiomata on twig. **(C)** Transverse section of conidioma. **(D)** Longitudinal section through conidioma. **(E)** Conidiogenous cells and conidia. **(F)** Alpha and beta conidia. Scale bars: 2 mm **(A)**; 500 µm **(B–D)**; 10 µm **(E, F)**.

***Diaporthe eres*** Nitschke, Pyrenomyc. Germ. 2: 245, 1870. **Figure 8**.

Synonyms are listed in Yang et al. (2018).

*Description*: Necrotic tissues on stems and branches of *Corylus heterophylla*. Sexual morph: not observed. Asexual morph: Stromata immersed in bark. Pycnidial stromata ostiolated, scattered or serried, discoid to conical, erumpent slightly through the bark surface at maturity, with single locule. Conceptacle absent. 140–380 (av. = 221, n = 30) µm in diameter. Ectostromatic disc brown to black with only one ostiole in center, discoid, circular to ovoid, 120–270 (av. = 163, n = 30) µm in diameter. Ostiole dark-grey, conspicuous, at the same level as the disc surface, 20–80 (av. = 56, n = 30) µm in diameter. Conidiogenous cells phialidic, cylindrical, terminal, 7.5–16.0 × 2.0–3.0 (av. = 12.2 ± 2.5 × 2.6 ± 0.3, n = 30) mm. Alpha conidia hyaline, aseptate, ellipsoidal, one guttulate at each end, 5.5–8.5 × 1.5–2.5 (av. = 6.5 ± 0.6 × 2.1 ± 0.2, n = 50) μm, L/W ratio, 2.58–4.75 (av. = 3.14 ± 0.41). Beta conidia hyaline, lanceolate to linear, 12.5–30.5 × 1.0–1.5 (av. = 23.9 ± 4.0 × 1.2 ± 0.2, n = 50) μm, L/W ratio 8.99–32.06 (av. = 19.91 ± 4.23).

*Culture characteristics*: Colonies with felty aerial mycelium, white, changing to compact at center later and sparse at surrounding. Growing up to 45 mm after three days incubation. Conidiomata sparse, black, distributed irregularly (**Figure 9F**).

*Specimens examined*: CHINA, Beijing City, Huairou District, Forestry Center of Beijing University of Agriculture, 116°26′35.53″E, 40°52′47.18″N, from branches of *Corylus heterophylla*, June, 2019, *H. Gao* & *X.L. Fan* (BJFC CF20210119), living culture CFCC 53999 and CFCC 54714; CHINA, Beijing City, Huairou District, Forestry Center of Beijing University of Agriculture, 116°26′41.03″E, 40°52′29.63″N, from stems of *C. heterophylla*, June, 2019, *H. Gao* & *X.L. Fan* (BJFC CF20210120), living culture CFCC 54715 to 54716.

*Notes*: *Diaporthe eres* were defined first by Nitschke (1870), collected from *Ulmus* sp. in Germany. Udayanga et al. (2014b) described them as a complex and provided a phylogram of seven genes. Phenotypic plasticity and host affiliations have been used for species identification of this complex, but have shown little significance (Udayanga et al., 2014a; Udayanga et al., 2014b; Udayanga et al., 2015; Du et al., 2016; Gao et al., 2016; Gao et al., 2017; Dissanayake et al., 2017). Fan et al. (2018) investigated this complex using a three (*cal*, *tef1-α*, and *tub2*) data matrix and further identified the species. *Diaporthe eres* were identified as pathogens on hazel trees in Oregon (Battilani et al., 2018). The current results indicate four strains of *D. eres* from hazel trees in China.

### Effects of Temperature, pH, and Carbon Source on Mycelial Growth

#### Effects on Mycelial Growth of *Cytospora corylina*

**Figure 10:**
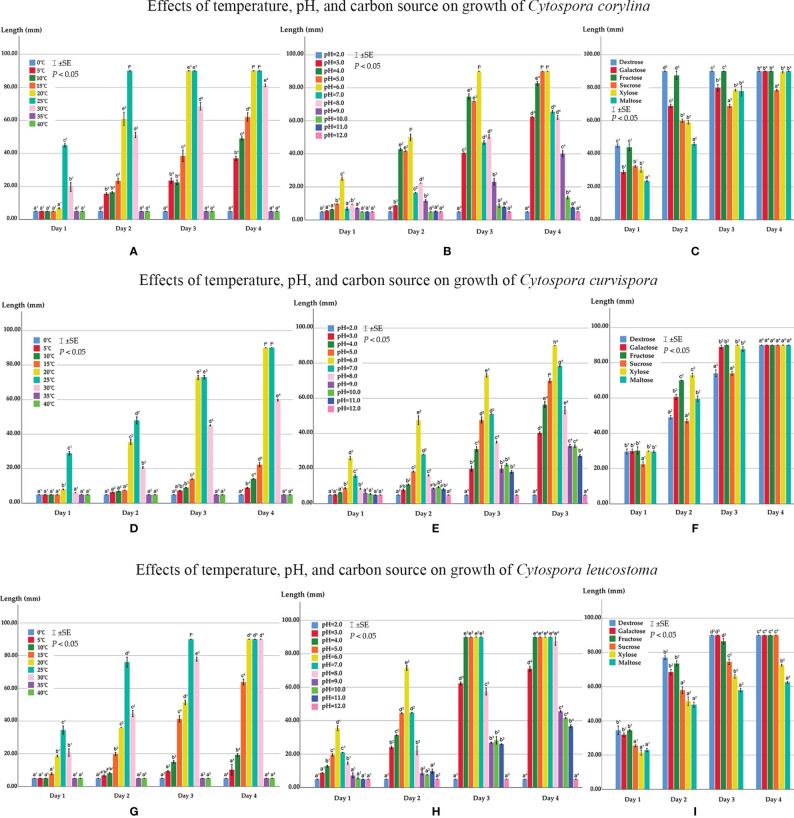
**(A)** Effects of temperature on growth of *Cytospora corylina*. **(B)** Effects of pH on growth of *Cytospora corylina*. **(C)** Effects of carbon source on growth of *Cytospora corylina*. **(D)** Effects of temperature on growth of *Cytospora curvispora*. **(E)** Effects of pH on growth of *Cytospora curvispora*. **(F)** Effects of carbon source on growth of *Cytospora curvispora*. **(G)** Effects of temperature on growth of *Cytospora leucostoma*. **(H)** Effects of pH on growth of *Cytospora leucostoma*. **(I)** Effects of carbon source on growth of *Cytospora leucostoma*. Bars represent ± SE. Mycelium length not connected by the same letter in a group are apparently different (*p <*0.05) for that condition.

*Cytospora corylina* (CFCC 54684) expressed high adaptability to the three conditions and fast growth rate. Colonies grew from 5 to 30°C but not at 0, 35, or 40°C after 96 h of incubation (**Figure 10A**). The maximal growth occurred at 25°C after 24 h, at which point colonies grew up to 45 mm diameter, reaching 90 mm after 48 h. After 72 h, colonies reached 90 mm at 20°C and 70 mm at 30°C, and after 96 h, the colony diameter reached 81 mm. Colonies grew on PDA in the pH range 3.0–11.0 but not at 2.0 or 12.0 (**Figure 10B**). Mycelium had the highest growth rate at pH 6.0, reaching diameters of 25 mm after 24 h and 90 mm after 72 h, followed by pH 4.0 and 8.0, which resulted in colonies of 75 mm and 51 mm diameters, respectively, after 72 h. However, on the fourth day, colonies showed fast growth in pH 5.0, reaching up to 90 mm diameter. The slowest growth occurred at pH 11.0, with colonies reaching only seven millimeters in diameter after four days of incubation. In general, *C. corylina* is more suitable for weakly acidic conditions ranging from pH 4.0 to 7.0.

*Cytospora corylina* grew on all six tested carbon sources (**Figure 10C**). The mycelia grew faster in dextrose- and fructose-supplemented media after 24 h than in media supplemented with the other carbon sources. After 48 h, mycelium reached 90 mm diameter on dextrose-supplemented medium, followed by fructose-supplemented medium; maltose-supplemented medium showed the least efficiency in terms of growth. The difference in the utilization of sucrose and other carbon sources gradually became apparent after 72 h: growth in sucrose-supplemented medium was significantly lower than that in media supplemented with the other carbon sources, all of which resulted in mycelium reaching 90 mm diameter after 96 h.

#### Effects on Mycelial Growth of *Cytospora curvispora*

Colonies of *Cytospora curvispora* (CFCC 54000) in the current study grew from 5 to 30°C but not at 0, 35, or 40°C after four days of dark incubation (**Figure 10D**). We observed the maximum total growth at 25°C after 24 h, and colonies grew up to 29 mm, followed by growth at 20 and 30°C. After 72 h, colonies reached 73 mm at 20 and 25°C and showed colony diameters of 90 mm after 96 h. *Cytospora curvispora* was highly adaptable to pH, the colonies of which could grow at pH 3.0–11.0 but not at 2.0 or 12.0 (**Figure 10E**). Growth was the fastest at pH 6.0, where mycelium reached 48 mm after 48 h and 90 mm after 96 h, followed by pH 7.0 in which a colony diameter of 78 mm was achieved. After 96 h, mycelial growth was the slowest at pH 11.0, reaching only 27 mm in diameter after four days of incubation.

*Cytospora curvispora* could utilize all six carbon sources (**Figure 10F**). After 24 h, the utility of the six different carbon sources was almost the same, except for sucrose. However, the difference in the utilization of these carbon sources gradually became apparent after 48 h. Growth on xylose- and fructose-supplemented media were significantly greater than that on the other carbon source-supplemented media, showing no difference after 96 h, with colonies of 90 mm diameter.

#### Effects on Mycelial Growth of *Cytospora leucostoma*

*Cytospora leucostoma* (CFCC 54680) expressed high adaptability to the three conditions. Colonies of it in the current study grew from 5–30°C but not at 0, 35, or 40°C after four days of incubation (**Figure 10G**). The maximum growth rate was observed at 25°C. Colonies reached 90 mm diameter at 25 and 30°C and nearly 80 mm in diameter after 72 h. At 20, 25, and 30°C, mycelium grew up to 90 mm after 96 h. Colonies could grow at a pH range of 3.0–11.0 but not at 2.0 and 12.0 (**Figure 10H**). We observed the maximum growth rate at pH 6.0, with diameters reaching 35 mm after 24 h and 72 mm after 48 h, followed by pH 5.0 and 7.0, both of which resulted in diameters of 45 mm after 48 h. After 96 h, colonies grew up to 90 mm diameter under pH 4.0, 5.0, 6.0, and 7.0 and reached 88 mm at pH 8.0.

*Cytospora leucostoma* could utilize six carbon sources (**Figure 10I**). The utility of dextrose, galactose, and fructose was greater than that of sucrose, xylose, and maltose after 96 h of incubation. After 72 h, mycelia growing on dextrose- and galactose-supplemented media were the first to reach 90 mm diameter, followed by colonies growing on fructose- and sucrose-supplemented media. Colonies grew slower on maltose-supplemented medium, reaching only 63 mm after four days.

#### Effects on Mycelial Growth of *Diaporthe corylicola*

**Figure 11:**
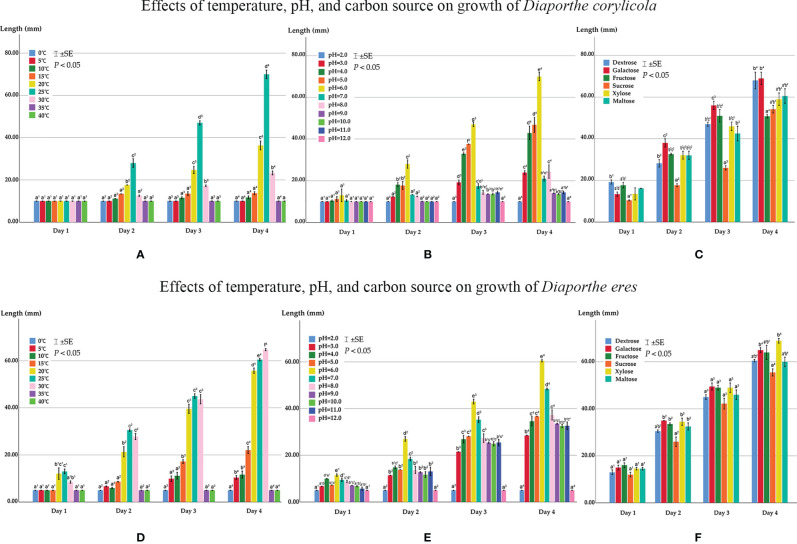
**(A)** Effects of temperature on growth of *Diaporthe corylicola*. **(B)** Effects of pH on growth of *Diaporthe corylicola*. **(C)** Effects of carbon source on growth of *Diaporthe corylicola.* **(D)** Effects of temperature on growth of *Diaporthe eres*. **(E)** Effects of pH on growth of *Diaporthe eres*. **(F)** Effects of carbon source on growth of *Diaporthe eres.* Bars represent ± SE. Mycelium length not connected by the same letter in a group are apparently different (*p <*0.05) for that condition.

Colonies of *Diaporthe corylicola* (CFCC 53986) in the current study grew on 10–30°C (**Figure 11A**). The maximum growth rate occurred at 25°C, but almost no growth was observed on the first day. *Diaporthe corylicola* grew slowly, and the colony diameter only reached 35 mm at 25°C after 96 h, followed by 18 mm diameter at 20°C. Colonies grew on PDA in the pH range of 3.0–11.0 but not at 2.0 or 12.0 (**Figure 11B**). The maximum mycelial growth was achieved at pH 6.0, reaching 35 mm after 96 h, followed by pH 5.0 and 4.0, and growth was slower at the other pH conditions. All six carbon sources tested could be metabolized by *D. corylicola* (**Figure 11C**). In the first three days, no significant difference was observed among the growth of colonies in media supplemented with all carbon sources, except sucrose. After 96 h of incubation, the utilization of dextrose and galactose was apparently greater, while that of the other four carbon sources reached nearly the same level.

#### Effects on Mycelial Growth of *Diaporthe eres*

Colonies of *Diaporthe eres* (CFCC 53999) grew at 5–30°C but not at 0°C, 35°C, or 40°C after four days. At 25°C, the maximum growth rate was achieved at 24 h, while at 30°C, the maximum growth rate was achieved at 96 h (**Figure 11D**). Colony growth was comparable at 20–30°C. After 96 h, colonies reached 65 mm diameter at 30°C, followed by 61 mm and 56 mm, respectively, at 25 and 20°C, while at the other temperatures, a slow growth rate was observed with no more than 25 mm diameter. *Diaporthe eres* were highly adaptable to pH, the colonies of which grew on PDA at a pH range of 3.0–11.0 but not at 2.0 or 12.0 (**Figure 11E**). Mycelium grew the fastest at pH 6.0, reaching 61 mm after 96 h, followed by pH 7.0, reaching 43 mm diameter after 96 h. In the other pH environments, although the growth rate was quite different from that at pH 6.0 and 7.0, it was maintained at a relatively high level.

All six carbon sources were utilized by *D. eres* (**Figure 11F**). After 24 h, the mycelial length was the same for colonies grown on media containing the six different carbon sources and was maintained at a similar level over 96 h. The medium containing xylose was utilized the best, and that containing sucrose was utilized slightly less than media containing the other carbon sources.

## Discussion

### Identification of *Cytospora* Species

Members of *Cytospora* have been reported as plant pathogens in forest and urban trees, including Anacardiaceae, Elaeagnaceae, Fabaceae, Juglandaceae, Myrtaceae, Rosaceae, Salicaceae, and Ulmaceae (Ehrenberg, 1818; Adams et al., 2002; Adams et al., 2005; Adams et al., 2006; Mehrabi et al., 2011; Wang et al., 2011; Fan et al., 2014a; Fan et al., 2014b; Fan et al., 2015a; Fan et al., 2015b; Lawrence et al., 2017; Fan et al., 2020). Previously, the identification of *Cytospora* relied on morphology and host affiliation. However, host affiliation is not always stable, and some species have similar morphology and uninformative illustrations and descriptions (Teng, 1963; Tai, 1979; Wei, 1979; Adams et al., 2002; Adams et al., 2005; Wang et al., 2011; Fan et al., 2014a; Fan et al., 2014b; Lawrence et al., 2017; Fan et al., 2020; Pan et al., 2020). ITS sequences were first used in *Cytospora* species identification by Adams et al. (2002), with six *Cytospora* groups suggested. Twenty-eight *Cytospora* species have been described from *Eucalyptus*, and 144 strains representing 20 species of *Cytospora* were subsequently collected from Iran (Adams et al., 2005; Fotouhifar et al., 2010). Recently, morphology combined with multi-locus phylogeny has been used for species identification, and has revealed many cryptic species (Fan et al., 2014a; Fan et al., 2015a; Fan et al., 2015b; Lawrence et al., 2017; Lawrence et al., 2018; Norphanphoun et al., 2018; Shang et al., 2020).

Fan et al. (2020) used six genes (ITS, LSU, *act*, *rpb2*, *tef1-α*, and *tub2*) to summarize 52 *Cytospora* species in China. Nevertheless, hidden fungal diversity has been revealed continuously in some special plant hosts (Pan et al., 2020; Pan et al., 2021). The present study revealed three species associated with *Corylus heterophylla*, i.e., *Cytospora corylina*, *C. curvispora*, and *C. leucostoma*. As the LSU gene is only available for a few species, we adapted a five-gene sequence of ITS, *act*, *rpb2*, *tef1-α*, and *tub2* in the phylogeny analyses. Moreover, although *C. leucostoma* has been reported as a common species that causes canker in plants of Rosaceae, we have a poor understanding of its host specificity and pathogenicity (Pan et al., 2020). This study represents an attempt to enrich the study of *Cytospora* in China.

### Identification of *Diaporthe* Species

*Diaporthe* was established by Nitschke (1870) and has been extensively studied by Udayanga et al. (2011; 2012a) in recent years. Species of this genus, including endophytes, saprobes, and plant pathogens, are widely distributed in natural ecosystems (Udayanga et al., 2011; Udayanga et al., 2012a).

The species were initially determined based on host affiliations and morphological features (Aa et al., 1990). However, in terms of phylogenetic relationships, morphology and host association usually showed little siginificance (Brayford, 1990; Rehner and Uecker, 1994; Udayanga et al., 2014a; Udayanga et al., 2014b; Udayanga et al., 2015). Molecular techniques have been utilized in the latest taxonomic methods to define *Diaporthe* species (Santos and Phillips, 2009; Santos et al., 2010; Udayanga et al., 2011; Udayanga et al., 2012a; Udayanga et al., 2012b; Gomes et al., 2013; Udayanga et al., 2014a; Udayanga et al., 2014b; Udayanga et al., 2015). Since these revolutionary studies, more than 50 novel *Diaporthe* species have been identified in China (Huang et al., 2013; Huang et al., 2015; Gao et al., 2016; Gao et al., 2017; Yang et al., 2017a; Yang et al., 2017b; Yang et al., 2018; Fan et al., 2018; Yang et al., 2020). New records and species have been reported on the basis of molecular evidence (Rossman et al., 2015; Dissanayake et al., 2017; Guarnaccia and Crous, 2017; Perera et al., 2018; Tibpromma et al., 2018; Wanasinghe et al., 2018; Wrona et al., 2020).

In the current study, based on multi-locus sequences (ITS, *cal*, *his3*, *tef1-α*, and *tub2*), we identified two *Diaporthe* species associated with hazelnut. The known species, *D. eres*, has been widely reported as a plant pathogen. The other was identified as a new species, *D. corylicola*, with highly supported clades and holomorphic morphology.

### Fungal Diversity Associated With Hazelnut

A great number of fungal pathogens associated with many *Corylus* species, especially *C. avellana*, have been identified based on molecular data and morphological characteristics in recent studies (Pinkerton et al., 1992; Chen et al., 2007; Guerrero and Pérez, 2013a; Guerrero and Pérez, 2013b; Linaldeddu et al., 2016; Wiman et al., 2019; Yang et al., 2020; Zhu et al., 2020). A broad list of fungi has been reported on different parts of hazel trees. For example, *Anisogramma*, *Anthostoma*, *Diaporthe*, *Diaporthella*, *Diplodia*, *Dothiorella*, and *Gnomoniopsis* are common fungi that inhabit branches in hazel trees in America, Chile, Italy, and Turkey (Gottwald and Cameron, 1980; Guerrero and Pérez, 2013a; Linaldeddu et al., 2016). *Alternaria*, *Aspergillus*, *Botryosphaeria*, *Colletotrichum*, *Diaporthe*, *Fusarium*, *Pestalotiopsis*, and *Phoma* are often isolated from fruits, especially in Turkey and the Caucasus region (Sezer and Dolar, 2016; Battilani et al., 2018; Arciuolo et al., 2020; Arciuolo et al., 2021). Although little information is available, *Cytospora corylicola* generally occurs in hazelnut growing areas in Europe (Salerno, 1961). These studies indicate great similarities among fungi in hazelnut cultivating areas in different geographical locations and environmental conditions. A recent report introduced *Elsinoë coryli* (*Sphaceloma coryli*) as a re-emerging pathogen on hazel trees in southern Italy, and nine other species were collected from symptomatic branches in Sardinia (Italy), causing serious economic losses (Lamichhane et al., 2014; Linaldeddu et al., 2016; Minutolo et al., 2016; Fan et al., 2017). Thus, several studies have confirmed the high fungal diversity associated with European hazelnut.

In the current study, 51 strains were isolated from the stems and branches of *Corylus heterophylla* collected from Beijing, China. Among them, 14 were identified as *Cytospora* and the other 37 as *Diaporthe*. Among them, *Cytospora corylina* and *C. leucostoma* were only isolated from branches and other three species were observed both on stems and branches. These results indicate that the diversity of fungi associated with hazelnut canker and dieback disease is greater than previously recognized. All species in this study, except *Diaporthe eres*, were discovered for the first time on hazel trees. Previously, *Cytospora coryli* and *Diaporthe coryli* were reported as pathogens on branches of *Corylus mandshurica*, which is related to and generally grows naturally alongside *C. heterophylla* (Yang et al., 2020; Zhu et al., 2020). Thus, we speculate that *C. heterophylla* has great potential to be infected by *Cytospora coryli* and *Diaporthe coryli*. Although many *Cytospora* and *Diaporthe* species are endophytes and saprobes, it is still unclear whether opportunistic species would transform into pathogens on new hosts or in different environmental conditions, or adapt to climate change (Wang et al., 2020). Furthermore, *Cytospora leucostoma* and *Diaporthe eres* are plant pathogens that have been collected from a wide range of woody hosts, and *Diaporthe* species have been reported as the main cause of hazelnut defects in the Caucasus region (Battilani et al., 2018; Wiman et al., 2019). Thus, extensive investigations and pathogenicity tests on the five species need a further study.

### Optimum Environment for Culturing Isolates

Most fungi can grow at 10–35°C, with the most suitable range between 20 and 30°C. The most suitable pH for mycelial growth is 5.0–6.5 (Elfar et al., 2013). For instance, the optimal growth temperature of *Diaporthe* sp. is 22°C (Strausbaugh and Dugan, 2017), and that of *Cytospora hadianensis* is 19.8°C (Zhou et al., 2020). Helton and Konicek (1962) isolated six *Cytospora* species and indicated that the optimum pH is approximately 4.5, with a temperature of 20–35°C. Similar results were obtained in this study; all five species tested grew on PDA at 5–30°C and a pH of 3.0–11.0, with an optimum temperature of 20–30°C and pH value of 4.0–7.0. However, *Cytospora corylina* incubated at 15 and 20°C showed special growth characteristics on PDA. It formed very fine mycelial strands, which radiated outward from the central portion of the plate, and did not have the same physical appearance as the rest of the mycelium. The special growth characteristics of *Cytospora* species have been observed by Konicek and Helton (1962a), who provided a reference to include these fine strands in the measurement. As a result, the optimum conditions for *Cytospora* species were 20–30°C and pH 4.0–7.0, and those for *Diaporthe* species were 20–30°C and pH 5.0–7.0. If one unusual factor alone is removed, such as the aberrant marginal growth of *C. corylina* on PDA, optimum temperature for *Cytospora* is 25–30°C.

The six carbon sources tested in the current study were efficiently utilized, although the utilization of sucrose by *Cytospora corylina*, *C. curvispora*, and *Diaporthe eres* was less than that of the other sources. Zhou et al. (2020) reported that utilization of galactose by *Cytospora hadianensis* is low, and Zhao et al. (2019) reported that the utilization of xylose by *Lasiodiplodia vaccinii* and *L*. *theobromae* is the lowest. The best overall growth results of the six *Cytospora* species tested were obtained with maltose. However, none of the species in our study had the best overall growth on maltose media, but all had a high ability to use galactose and xylose, except *Cytospora leucostoma* for xylose.

Cultured isolates of different species differ subtly in mycelial growth and pathogenicity under a given condition, and one species may have different characteristics under different conditions (Konicek and Helton, 1962a; Konicek and Helton, 1962b; Wang et al., 2020). The different growth characteristics and high adaptability to the environmental conditions of these *Cytospora* and *Diaporthe* species seem to justify their widespread occurrence. Biological characterization of these strains, combined with their host distribution, may be used for species identification and distribution prediction. However, as there are currently few details regarding pathogen biology, it is necessary to evaluate the effects of environmental conditions, such as temperature, pH, and carbon sources, on mycelium growth and pathogenicity.

## Data Availability Statement

The datasets presented in this study can be found in online repositories. The names of the repository/repositories and accession number(s) can be found in the article/**Supplementary Material**.

## Author Contributions

All authors have made extensive contributions to the work presented in the article. XF and CT: contributed to conception of the experiment. HG and MP: completed the experiment. HG: conducted the data analyses. HG: wrote the original manuscript. XF: reviewed and edited the draft. All authors contributed to the article and approved the submitted version.

## Funding

This research was funded by the National Natural Science Foundation of China (31670647), and College Student Research and Career-Creation Program of Beijing (202010022256).

## Conflict of Interest

The authors declare that the research was conducted in the absence of any commercial or financial relationships that could be construed as a potential conflict of interest.
