# Supplementary material for: Cytospora and Diaporthe Species Associated With Hazelnut Canker and Dieback in Beijing, China
Source: Front Cell Infect Microbiol. 2021 Aug 2;11:664366. doi: 10.3389/fcimb.2021.664366 (PMC8366500; doi:10.3389/fcimb.2021.664366)
Supplement: Supplementary file 1 [file Table_1.docx]

**Supplymentary Table 1.** Genes used in this study with PCR primers and process.

| **Genus** | **Locus** | **PCR primers** | **PCR:thermal cycles: (Annealing temp. in bold)** | **Reference** |
| --- | --- | --- | --- | --- |
| *Cytospora* | ITS | ITS1/ITS4 | (95 °C: 30 s, **51 °C**: 30 s, 72 °C: 1 min) × 35 cycles | White et al., 1990 |
|  | *act* | ACT-512F/ ACT-783R | (95 °C: 45 s, **55 °C**: 45 s, 72 °C: 1 min) × 35 cycles | Carbone and Kohn, 1999 |
|  | *rpb2* | RPB2-5F/RPB2-7cR | (95 °C: 30 s, **52 °C**: 1 min, 72 °C: 1 min) × 35 cycles | Liu et al., 1999 |
|  | *tef1-α* | EF1-728F/EF1-1251R | (95 °C: 15 s, **55 °C**: 20 s, 72 °C: 1 min) × 35 cycles | Carbone and Kohn, 1999 |
|  | *tub2* | Bt2a/Bt2b | (95 °C: 30 s, **55 °C**: 30 s, 72 °C: 1 min) × 35 cycles | Glass and Donaldson, 1995 |
| *Diaporthe* | ITS | ITS1/ITS4 | (95 °C: 30 s, **51 °C**: 30 s, 72 °C: 1 min) × 35 cycles | White et al., 1990 |
|  | *cal* | CAL228F/CAL737R | (95 °C: 15 s, **55 °C**: 20 s, 72 °C: 1 min) × 35 cycles | Carbone and Kohn, 1999 |
|  | *his3* | CYLH3F/H3-1b | (95 °C: 30 s, **58 °C**: 30 s, 72 °C: 1 min) × 35 cycles | Crous et al., 2004  Glass and Donaldson, 1995 |
|  | *tef1-α* | EF1-728F/EF1-986R | (95 °C: 15 s, **55 °C**: 20 s, 72 °C: 1 min) × 35 cycles | Carbone and Kohn, 1999 |
|  | *tub2* | Bt2a/Bt2b | (95 °C: 30 s, **55 °C**: 30 s, 72 °C: 1 min) × 35 cycles | Glass & Donaldson, 1995 |
