# Supplementary material for: Cytospora and Diaporthe Species Associated With Hazelnut Canker and Dieback in Beijing, China
Source: Front Cell Infect Microbiol. 2021 Aug 2;11:664366. doi: 10.3389/fcimb.2021.664366 (PMC8366500; doi:10.3389/fcimb.2021.664366)
Supplement: Supplementary file 3 [file Table_3.docx]

**Supplymentary Table 3.** Strains of *Diaporthe* used in the molecular analyses in this study.

| **Species** | **Strain^2^** | **Host** | **Origin** | **GenBank accession numbers** | | | | |
| --- | --- | --- | --- | --- | --- | --- | --- | --- |
|  |  |  |  | *cal* | *his3* | ITS | *tef1-α* | *tub2* |
| *Diaporthe acaciigena* | CBS 129521^T^ | *Acacia retinodes* | Australia | KC343247 | KC343489 | KC343005 | KC343731 | KC343973 |
| *Diaporthe acericola* | MFLUCC 17-0956^T^ | *Acer negundo* | Italy | KY964137 | NA | KY964224 | KY964180 | KY964074 |
| *Diaporthe acerigena* | CFCC 52554^T^ | *Acer tataricum* | China | MH121413 | MH121449 | MH121489 | MH121531 | NA |
| *Diaporthe acerigena* | CFCC 52555 | *Acer tataricum* | China | MH121414 | MH121450 | MH121490 | MH121532 | NA |
| *Diaporthe acutispora* | LC6161 | *Coffea* sp. | China | KX999274 | KX999235 | KX986764 | KX999155 | KX999195 |
| *Diaporthe alangii* | CFCC 52556^T^ | *Alangium kurzii* | Beijing, China | MH121415 | MH121451 | MH121491 | MH121533 | MH121573 |
| *Diaporthe alangii* | CFCC 52557 | *Alangium kurzii* | Beijing, China | MH121416 | MH121452 | MH121492 | MH121534 | MH121574 |
| *Diaporthe albosinensis* | CFCC 53066 | *Betula albosinensis* | Shaanxi, China | MK442979 | MK443004 | MK432659 | MK578133 | MK578059 |
| *Diaporthe albosinensis* | CFCC 53067 | *Betula albosinensis* | Shaanxi, China | MK442980 | MK443005 | MK432660 | MK578134 | MK578060 |
| *Diaporthe alleghaniensis* | CBS 495.72^T^ | *Betula alleghaniensis* | Canada | MH121426 | MH121462 | MH121502 | MH121544 | MH121584 |
| *Diaporthe alnea* | CBS 146.46^T^ | *Alnus* sp. | Netherlands | KC343250 | KC343492 | KC343008 | KC343734 | KC343976 |
| *Diaporthe ambigua* | CBS 114015 | *Pyrus communis* | South Africa | KC343252 | KC343494 | KC343010 | KC343736 | KC343978 |
| *Diaporthe ampelina* | STE-U 2660 | *Vitis vinifera* | France | AY745026 | NA | NA | AY745056 | NA |
| *Diaporthe amygdali* | CBS 126679^T^ | *Prunus dulcis* | Portugal | KC343264 | KC343506 | MH864208 | KC343748 | KC343990 |
| *Diaporthe anacardii* | CBS 720.97^T^ | *Anacardium occidentale* | East Africa | KC343266 | KC343508 | KC343024 | KC343750 | KC343992 |
| *Diaporthe angelicae* | CBS 111592^T^ | *Heracleum sphondylium* | Austria | KC343269 | KC343511 | KC343027 | KC343753 | KC343995 |
| *Diaporthe apiculatum* | CFCC 53068 | *Rhus chinensis* | China | MK442973 | MK442998 | MK432651 | MK578127 | MK578054 |
| *Diaporthe apiculatum* | CFCC 53069 | *Rhus chinensis* | China | MK44297 | MK442999 | MK432652 | MK578128 | MK578055 |
| *Diaporthe apiculatum* | LC3418^T^ | *Camellia sinensis* | China | NA | NA | KP267896 | KP267970 | KP293476 |
| *Diaporthe aquatica* | IFRDCC 3051^T^ | *Aquatic habitat* | China | NA | NA | JQ797437 | NA | NA |
| *Diaporthe arctii* | DP0482^T^ | *Arctium lappa* | Austria | KJ612133 | KJ659218 | KJ590736 | KJ590776 | KJ610891 |
| *Diaporthe arecae* | CBS 161.64^T^ | *Areca catechu* | India | KC343274 | KC343516 | KC343032 | KC343758 | KC344000 |
| *Diaporthe arengae* | CBS 114979^T^ | *Arenga engleri* | Hong Kong | KC343276 | KC343518 | MF773664 | KC343760 | KC344002 |
| *Diaporthe aseana* | MFLUCC 12-0299a | Unknown dead leaf | Thailand | KT459464 | NA | KT459414 | KT459448 | KT459432 |
| *Diaporthe asheicola* | CBS 136967 | *Vaccinium ashei* | Chile | KJ160542 | NA | KJ160562 | KJ160594 | KJ160518 |
| *Diaporthe aspalathi* | CBS 117169^T^ | *Aspalathus linearis* | South Africa | KC343278 | KC343520 | KC343036 | KC343762 | KC344004 |
| *Diaporthe australafricana* | CBS 111886^T^ | *Vitis vinifera* | Australia | KC343280 | KC343522 | KC343038 | KC343764 | KC344006 |
| *Diaporthe baccae* | CBS 136972^T^ | *Vaccinium corymbosum* | Italy | MG281695 | MF418264 | MK370623 | KJ160597 | MF418509 |
| *Diaporthe batatas* | CBS 122.21^T^ | *Ipomoea batatas* | USA | KC343282 | KC343524 | KC343040 | KC343766 | KC344008 |
| *Diaporthe bauhiniae* | CFCC 53071 | *Bauhinia purpurea* | China | MK442970 | MK442995 | MK432648 | MK578124 | MK578051 |
| *Diaporthe bauhiniae* | CFCC 53072 | *Bauhinia purpurea* | China | MK442971 | MK442996 | MK432649 | MK578125 | MK578052 |
| *Diaporthe bauhiniae* | CFCC 53073 | *Bauhinia purpurea* | China | MK442972 | MK442997 | MK432650 | MK578126 | MK578053 |
| *Diaporthe beilharziae* | BRIP 54792^T^ | *Indigofera australis* | Australia | NA | NA | JX862529 | JX862535 | KF170921 |
| *Diaporthe benedicti* | SBen914 | *Diaporthe benedicti* | USA | KM669862 | NA | KM669929 | KM669785 | NA |
| *Diaporthe betulae* | CFCC 50469 | *Betula platyphylla* | China | KT732997 | KT732999 | KT732950 | KT733016 | KT733020 |
| *Diaporthe betulae* | CFCC 50470 | *Betula platyphylla* | China | KT732998 | KT733000 | KT732951 | KT733017 | KT733021 |
| *Diaporthe betulicola* | CFCC 51128^T^ | *Betula albo-sinensis* | China | KX024659 | KX024661 | KX024653 | KX024655 | KX024657 |
| *Diaporthe betulicola* | CFCC 51129 | *Betula albo-sinensis* | China | KX024660 | KX024662 | KX0246554 | KX0246556 | KX024658 |
| *Diaporthe betulina* | CFCC 52560 | *Betula albo-sinensis* | China | MH121419 | MH121455 | MH121495 | MH121537 | MH121577 |
| *Diaporthe betulina* | CFCC 52561 | *Betula albo-sinensis* | China | MH121420 | MH121456 | MH121496 | MH121538 | MH121578 |
| *Diaporthe bicincta* | CBS 121004^T^ | *Juglans* sp. | USA | KC343376 | KC343618 | KC343134 | KC343860 | KC344102 |
| *Diaporthe biconispora* | ZJUD62 | *Citrus maxima* | Fujian, China | NA | KJ490539 | KJ490597 | KJ490476 | KJ490418 |
| *Diaporthe biguttulata* | ZJUD47 | *Citrus limon* | Yunnan, China | NA | KJ490524 | KJ490582 | KJ490461 | KJ490403 |
| *Diaporthe biguttusis* | CGMCC 3.17081 | *Lithocarpus glabra* | China | NA | NA | KF576282 | KF576257 | KF576306 |
| *Diaporthe bohemiae* | CBS 143347^T^ | *Vitis vinifera* | Czech Republic | MG281710 | MG281361 | MK300012 | MG281536 | MG281188 |
| *Diaporthe brasiliensis* | CBS 133183^T^ | *Aspidosperma tomentosum* | Brazil | KC343284 | KC343526 | KC343042 | KC343768 | KC344010 |
| *Diaporthe caatingaensis* | URM7485 | *Tacinga inamoena* | Brazil | KY115598 | NA | KY085927 | KY115604 | KY115601 |
| *Diaporthe camptothecicola* | CFCC 51632 | *Camptotheca acuminata* | China | KY228877 | KY228881 | KY203726 | KY228887 | KY228893 |
| *Diaporthe canthii* | CPC 19740 | *Canthium inerme* | South Africa | NA | NA | JX069864 | NA | NA |
| *Diaporthe caryae* | CFCC 52563^T^ | *Carya illinoinensis* | Beijing, China | MH121422 | MH121458 | MH121498 | MH121540 | MH121580 |
| *Diaporthe caryae* | CFCC 52564 | *Carya illinoinensis* | Beijing, China | MH121423 | MH121459 | MH121499 | MH121541 | MH121581 |
| *Diaporthe cassines* | CPC 21916^T^ | *Cassine peragua* | South Africa | NA | NA | KF777155 | KF777244 | NA |
| *Diaporthe caulivora* | CBS 127268^T^ | *Glycine max* | Croatia | KC343287 | KC343529 | MH864501 | KC343771 | KC344013 |
| *Diaporthe celastrina* | CBS 139.27^T^ | *Celastrus* sp. | USA | KC343289 | KC343531 | KC343047 | KC343773 | KC344015 |
| *Diaporthe celeris* | CBS 143349^T^ | *Vitis vinifera* | United Kingdom | MG281712 | MG281363 | MG281017 | MG281538 | MG281190 |
| *Diaporthe cercidis* | CFCC 52565^T^ | *Cercis chinensis* | China | MH121424 | MH121460 | MH121500 | NA | MH121582 |
| *Diaporthe cercidis* | CFCC 52566 | *Cercis chinensis* | China | MH121425 | MH121461 | MH121501 | NA | MH121583 |
| *Diaporthe chamaeropis* | CBS 454.81 | *Chamaerops humilis* | Greece | KC343290 | KC343532 | KC343048 | KC343774 | KC344016 |
| *Diaporthe charlesworthii* | BRIP 54884m^T^ | *Rapistrum rugostrum* | Australia | NA | NA | KJ197288 | KJ197250 | KJ197268 |
| *Diaporthe chensiensis* | CFCC 52567^T^ | *Abies chensiensis* | China | MH121426 | MH121462 | MH121502 | MH121544 | MH121584 |
| *Diaporthe chensiensis* | CFCC 52568 | *Abies chensiensis* | China | MH121427 | MH121463 | MH121503 | MH121545 | MH121585 |
| *Diaporthe cichorii* | MFLUCC 17-1023^T^ | *Cichorium intybus* | Italy | KY964133 | NA | KY964220 | KY964176 | KY964104 |
| *Diaporthe cinnamomi* | CFCC 52569^T^ | *Cinnamomum* sp*.* | Beijing, China | NA | MH121464 | MH121504 | MH121546 | MH121586 |
| *Diaporthe cinnamomi* | CFCC 52570 | *Cinnamomum* sp*.* | Beijing, China | NA | MH121465 | MH121505 | MH121547 | MH121587 |
| *Diaporthe cissampeli* | CPC 27302^T^ | *Cissampelos capensis* | South Africa | NA | KX228366 | KX228273 | NA | KX228384 |
| *Diaporthe citri* | AR3405 | *Citrus* sp. | USA | KC843157 | KJ420881 | KC843311 | KC843071 | KC843187 |
| *Diaporthe citri* | CFCC 53079 | *Citrus sinensis* | China | MK574579 | MK574595 | MK573940 | MK574615 | MK574635 |
| *Diaporthe citri* | CFCC 53080 | *Citrus sinensis* | China | MK574580 | MK574596 | MK573941 | MK574616 | MK574636 |
| *Diaporthe citriasiana* | CGMCC 3.15224 | *Citrus unshiu* | China | KC357491 | KC490515 | JQ954645 | JQ954663 | KC357459 |
| *Diaporthe citrichinensis* | CGMCC 3.15225 | *Citrus* sp. | China | KC357494 | NA | JQ954648 | JQ954666 | NA |
| *Diaporthe collariana* | MFLU 17-2770^T^ | *Magnolia champaca* | Thailand | MG783042 | NA | MG806115 | MG783040 | MG783041 |
| *Diaporthe compactum* | LC3083^T^ | *Camellia sinensis* | China | NA | KP293508 | KP267854 | KP267928 | NA |
| *Diaporthe conica* | CFCC 52571^T^ | *Alangium chinense* | China | MH121428 | MH121466 | MH121506 | MH121548 | MH121588 |
| *Diaporthe conica* | CFCC 52572 | *Alangium chinense* | China | MH121429 | MH121467 | MH121507 | MH121549 | MH121589 |
| *Diaporthe convolvuli* | CBS 124654^T^ | *Convolvulus arvensis* | Turkey | KC343296 | KC343538 | KC343054 | KC343780 | KC344022 |
| *Diaporthe coryli* | CFCC 53083^T^* | *Corylus mandshurica* | Shaanxi, China | MK442981 | MK443006 | MK432661 | MK578135 | MK578061 |
| *Diaporthe coryli* | CFCC 53084* | *Corylus mandshurica* | Shaanxi, China | MK442982 | MK443007 | MK432662 | MK538176 | MK578062 |
| ***Diaporthe corylicola*** | **CFCC 53986**^T^ | ***Corylus heterophylla*** | **Beijing, China** | **MW836684** | **MW836717** | **MW839880** | **MW815894** | **MW883977** |
| ***Diaporthe corylicola*** | **CFCC 53987** | ***Corylus heterophylla*** | **Beijing, China** | **MW836685** | **MW836718** | **MW839867** | **MW815895** | **MW883978** |
| ***Diaporthe corylicola*** | **CFCC 53988** | ***Corylus heterophylla*** | **Beijing, China** | **MW836686** | **MW836719** | **MW839868** | **MW815896** | **MW883979** |
| ***Diaporthe corylicola*** | **CFCC 53989** | ***Corylus heterophylla*** | **Beijing, China** | **MW836687** | **MW836720** | **MW839869** | **MW815897** | **MW883980** |
| ***Diaporthe corylicola*** | **CFCC 53990** | ***Corylus heterophylla*** | **Beijing, China** | **MW836688** | **MW836721** | **MW839870** | **MW815898** | **MW883981** |
| ***Diaporthe corylicola*** | **CFCC 53991** | ***Corylus heterophylla*** | **Beijing, China** | **MW836689** | **MW836722** | **MW839871** | **MW815899** | **MW883982** |
| ***Diaporthe corylicola*** | **CFCC 53991** | ***Corylus heterophylla*** | **Beijing, China** | **MW836690** | **MW836723** | **MW839872** | **MW815900** | **MW883983** |
| ***Diaporthe corylicola*** | **CFCC 53993** | ***Corylus heterophylla*** | **Beijing, China** | **MW836691** | **MW836724** | **MW839873** | **MW815901** | **MW883984** |
| ***Diaporthe corylicola*** | **CFCC 53994** | ***Corylus heterophylla*** | **Beijing, China** | **MW836692** | **MW836725** | **MW839875** | **MW815902** | **MW883985** |
| ***Diaporthe corylicola*** | **CFCC 53995** | ***Corylus heterophylla*** | **Beijing, China** | **MW836693** | **MW836726** | **MW839876** | **MW815903** | **MW883986** |
| ***Diaporthe corylicola*** | **CFCC 53996** | ***Corylus heterophylla*** | **Beijing, China** | **MW836694** | **MW836727** | **MW839877** | **MW815904** | **MW883987** |
| ***Diaporthe corylicola*** | **CFCC 53997** | ***Corylus heterophylla*** | **Beijing, China** | **MW836695** | **MW836728** | **MW839878** | **MW815905** | **MW883988** |
| ***Diaporthe corylicola*** | **CFCC 53998** | ***Corylus heterophylla*** | **Beijing, China** | **MW836696** | **MW836729** | **MW839879** | **MW815906** | **MW883989** |
| ***Diaporthe corylicola*** | **CFCC 54696** | ***Corylus heterophylla*** | **Beijing, China** | **MW836697** | **MW836730** | **MW839881** | **MW815907** | **MW883990** |
| ***Diaporthe corylicola*** | **CFCC 54697** | ***Corylus heterophylla*** | **Beijing, China** | **MW836698** | **MW836731** | **MW839882** | **MW815908** | **MW883991** |
| ***Diaporthe corylicola*** | **CFCC 54698** | ***Corylus heterophylla*** | **Beijing, China** | **MW836699** | **MW836732** | **MW839883** | **MW815909** | **MW883992** |
| ***Diaporthe corylicola*** | **CFCC 54699** | ***Corylus heterophylla*** | **Beijing, China** | **MW836700** | **MW836733** | **MW839884** | **MW815910** | **MW883993** |
| ***Diaporthe corylicola*** | **CFCC 54700** | ***Corylus heterophylla*** | **Beijing, China** | **MW836701** | **MW836734** | **MW839885** | **MW815911** | **MW883994** |
| ***Diaporthe corylicola*** | **CFCC 54701** | ***Corylus heterophylla*** | **Beijing, China** | **MW836702** | **MW836735** | **MW839886** | **MW815912** | **MW883995** |
| ***Diaporthe corylicola*** | **CFCC 54702** | ***Corylus heterophylla*** | **Beijing, China** | **MW836703** | **MW836736** | **MW839887** | **MW815913** | **MW883996** |
| ***Diaporthe corylicola*** | **CFCC 54703** | ***Corylus heterophylla*** | **Beijing, China** | **MW836704** | **MW836737** | **MW839888** | **MW815914** | **MW883997** |
| ***Diaporthe corylicola*** | **CFCC 54704** | ***Corylus heterophylla*** | **Beijing, China** | **MW836705** | **MW836738** | **MW839874** | **MW815915** | **MW883998** |
| ***Diaporthe corylicola*** | **CFCC 54705** | ***Corylus heterophylla*** | **Beijing, China** | **MW836706** | **MW836739** | **MW839889** | **MW815916** | **MW883999** |
| ***Diaporthe corylicola*** | **CFCC 54706** | ***Corylus heterophylla*** | **Beijing, China** | **MW836707** | **MW836740** | **MW839890** | **MW815917** | **MW884000** |
| ***Diaporthe corylicola*** | **CFCC 54707** | ***Corylus heterophylla*** | **Beijing, China** | **MW836708** | **MW836741** | **MW839891** | **MW815918** | **MW884001** |
| ***Diaporthe corylicola*** | **CFCC 54708** | ***Corylus heterophylla*** | **Beijing, China** | **MW836709** | **MW836742** | **MW839892** | **MW815919** | **MW884002** |
| ***Diaporthe corylicola*** | **CFCC 54709** | ***Corylus heterophylla*** | **Beijing, China** | **MW836710** | **MW836743** | **MW839893** | **MW815920** | **MW884003** |
| ***Diaporthe corylicola*** | **CFCC 54710** | ***Corylus heterophylla*** | **Beijing, China** | **MW836711** | **MW836744** | **MW839894** | **MW815921** | **MW884004** |
| ***Diaporthe corylicola*** | **CFCC 54711** | ***Corylus heterophylla*** | **Beijing, China** | **MW836712** | **MW836745** | **MW839895** | **MW815922** | **MW884005** |
| ***Diaporthe corylicola*** | **CFCC 54712** | ***Corylus heterophylla*** | **Beijing, China** | **MW836713** | **MW836746** | **MW839880** | **MW815923** | **MW884006** |
| ***Diaporthe corylicola*** | **CFCC 54713** | ***Corylus heterophylla*** | **Beijing, China** | **MW836714** | **MW836747** | **MW839865** | **MW815924** | **MW884007** |
| ***Diaporthe corylicola*** | **CFCC 54944** | ***Corylus heterophylla*** | **Beijing, China** | **MW836715** | **MW836748** | **MW839866** | **MW815925** | **MW884008** |
| ***Diaporthe corylicola*** | **CFCC 54945** | ***Corylus heterophylla*** | **Beijing, China** | **MW836716** | **MW836749** | **MW839895** | **MW815926** | **MW884009** |
| *Diaporthe crotalariae* | CBS 162.33^T^ | *Crotalaria spectabilis* | USA | JX197439 | KC343540 | MH855395 | GQ250307 | KC344024 |
| *Diaporthe cucurbitae* | DAOM 42078^T^ | *Cucumis* sp*.* | Canada | NA | KM453212 | KM453210 | KM453211 | KP118848 |
| *Diaporthe cuppatea* | CBS 117499^T^ | *Aspalathus linearis* | South Africa | KC343299 | KC343541 | MH863021 | KC343783 | KC344025 |
| *Diaporthe cynaroidis* | CBS 122676^T^ | *Protea cynaroides* | South Africa | KC343300 | KC343542 | KC343058 | KC343784 | KC344026 |
| *Diaporthe cytosporella* | FAU461 | *Citrus limon* | Italy | KC843141 | NA | KC843307 | KC843116 | KC843221 |
| *Diaporthe diospyricola* | CPC 21169^T^ | *Diospyros whyteana* | South Africa | NA | NA | KF777209 | NA | NA |
| *Diaporthe discoidispora* | ZJUD89 | *Citrus unshiu* | Jiangxi, China | NA | KJ490566 | KJ490624 | KJ490503 | KJ490445 |
| *Diaporthe dorycnii* | MFLUCC 17-1015^T^ | *Dorycnium hirsutum* | Italy | NA | NA | KY964215 | KY964171 | KY964099 |
| *Diaporthe elaeagni-glabrae* | LC4802 | *Elaeagnus glabra* | China | KX999281 | KX999251 | KX986779 | KX999171 | KX999212 |
| *Diaporthe ellipicola* | CGMCC 3.17084^T^ | *Lithocarpus glaber* | China | NA | NA | KF576270 | KF576245 | KF576294 |
| *Diaporthe endophytica* | CBS 133811^T^ | *Schinus terebinthifolius* | Brazil | KC343307 | KC343549 | KC343065 | KC343791 | KC344033 |
| *Diaporthe eres* | AR5193^T^ | *Ulmus* sp. | Germany | KJ434999 | KJ420850 | KJ210529 | KJ210550 | KJ420799 |
| *Diaporthe eres* | CFCC 52575 | *Castanea mollissima* | China | NA | MH121470 | MH121510 | MH121552 | MH121592 |
| *Diaporthe eres* | CFCC 52576 | *Castanea mollissima* | China | MH121432 | MH121471 | MH121511 | MH121553 | MH121593 |
| *Diaporthe eres* | CFCC 52577 | *Acanthopanax senticosus* | China | MH121433 | MH121472 | MH121512 | MH121554 | MH121594 |
| *Diaporthe eres* | CFCC 52578 | *Sorbus* sp. | China | MH121433 | MH121473 | MH121513 | MH121555 | MH121595 |
| *Diaporthe eres* | CFCC 52579 | *Juglans regia* | China | NA | MH121474 | MH121514 | MH121556 | NA |
| *Diaporthe eres* | CFCC 52580 | *Melia azedarace* | China | NA | MH121475 | MH121515 | MH121557 | MH121596 |
| *Diaporthe eres* | CFCC 52581 | *Rhododendron simsii* | China | NA | MH121476 | MH121516 | MH121558 | MH121597 |
| ***Diaporthe eres*** | **CFCC 53999** | ***Corylus heterophylla*** | **Beijing, China** | **NA** | **MW836750** | **MW839898** | **MW815927** | **MW884010** |
| ***Diaporthe eres*** | **CFCC 54714** | ***Corylus heterophylla*** | **Beijing, China** | **NA** | **MW836751** | **MW839899** | **MW815928** | **MW884011** |
| ***Diaporthe eres*** | **CFCC 54715** | ***Corylus heterophylla*** | **Beijing, China** | **NA** | **MW836752** | **MW839900** | **MW815929** | **MW884012** |
| ***Diaporthe eres*** | **CFCC 54716** | ***Corylus heterophylla*** | **Beijing, China** | **NA** | **MW836753** | **MW839901** | **MW815930** | **MW884013** |
| *Diaporthe eucalyptorum* | CBS 132525^T^ | *Eucalyptus* sp*.* | China | NA | NA | MH305525 | NA | NA |
| *Diaporthe foeniculacea* | CBS 111553 | *Foeniculum vulgare* | Spain | KC343343 | KC343585 | MH854926 | KC343827 | KC344069 |
| *Diaporthe fraxini-angustifoliae* | BRIP 54781^T^ | *Fraxinus angustifolia* | Australia | KT459462 | NA | JX862528 | JX862534 | NA |
| *Diaporthe fraxinicola* | CFCC 52582^T^ | *Fraxinus chinensis* | China | MH121435 | NA | MH121517 | MH121560 | NA |
| *Diaporthe fraxinicola* | CFCC 52583 | *Fraxinus chinensis* | China | MH121436 | NA | MH121518 | MH121559 | NA |
| *Diaporthe fukushii* | MAFF 625034 | *Pyrus pyrifolia* | Japan | KJ435023 | KJ420868 | NA | NA | KJ420819 |
| *Diaporthe fusicola* | CGMCC 3.17087 | *Lithocarpus glabra* | China | KF576233 | NA | KF576281 | KF576256 | KF576305 |
| *Diaporthe ganjae* | CBS 180.91^T^ | *Cannabis sativa* | USA | KC343354 | KC343596 | KC343112 | KC343838 | KC344080 |
| *Diaporthe ganzhouensis* | CFCC 53087 | Unknown dead wood | China | MK442985 | MK443010 | MK432665 | MK578139 | MK578065 |
| *Diaporthe ganzhouensis* | CFCC 53088 | Unknown dead wood | China | MK442986 | MK443011 | MK432666 | MK578140 | MK578066 |
| *Diaporthe garethjonesii* | MFLUCC 12-0542a | Unknown dead leaf | Thailand | KT459470 | NA | KT459423 | KT459457 | KT459441 |
| *Diaporthe goulteri* | BRIP 55657a^T^ | *Helianthus annuus* | Australia | NA | NA | KJ197290 | KJ197252 | KJ197270 |
| *Diaporthe gulyae* | BRIP 54025^T^ | *Helianthus annuus* | Australia | NA | NA | NA | JN645803 | KJ197271 |
| *Diaporthe helianthi* | CBS 592.81^T^ | *Helianthus annuus* | Serbia | KC343357 | KC343599 | KC343115 | KC343841 | KC344083 |
| *Diaporthe helicis* | AR5211^T^ | *Hedera helix* | France | KJ435043 | KJ420875 | KJ210538 | KJ210559 | KJ420828 |
| *Diaporthe heterophyllae* | CPC 26215 | *Acacia heterophylla* | France | MG600218 | MG600220 | MG600222 | MG600224 | MG600226 |
| *Diaporthe hickoriae* | CBS 145.26^T^ | *Carya glabra* | USA | KC343360 | NA | KC343118 | KC343844 | KC344086 |
| *Diaporthe hispaniae* | CBS 143351^T^ | *Vitis vinifera* | Spain | MG281820 | MG281471 | MG281123 | MG281644 | MG281296 |
| *Diaporthe hongkongensis* | CBS 115448^T^ | *Dichroa febrifuga* | China | KC343361 | KC343603 | MK304388 | KC343845 | KC344087 |
| *Diaporthe incompleta* | LC6754 | *Camellia sinensis* | China | KX999289 | KX999265 | KX986794 | KX999186 | KX999226 |
| *Diaporthe inconspicua* | CBS 133813^T^ | *Maytenus ilicifolia* | Brazil | KC343365 | KC343607 | NA | KC343849 | KC344091 |
| *Diaporthe infecunda* | CBS 133812^T^ | *Schinus terebinthifolius* | Brazil | KC343368 | KC343610 | KC343126 | KC343852 | KC344094 |
| *Diaporthe isoberliniae* | CPC 22549 | *Isoberlinia angolensis* | Zambia | NA | NA | KJ869190 | NA | KJ869245 |
| *Diaporthe juglandicola* | CFCC 51134^T^ | *Juglans mandshurica* | China | KX024616 | KX024622 | KU985101 | KX024628 | KX024634 |
| *Diaporthe juglandigena* | CFCC 52584 | *Juglans regia* | Beijing, China | MH121437 | MH121477 | MH121519 | MH121561 | MH121598 |
| *Diaporthe juglandigena* | CFCC 52585 | *Juglans regia* | Beijing, China | MH121438 | MH121478 | MH121520 | MH121562 | MH121599 |
| *Diaporthe kadsurae* | CFCC 52586^T^ | *Kadsura longipedunculata* | Beijing, China | MH121439 | MH121479 | MH121521 | MH121563 | MH121600 |
| *Diaporthe kadsurae* | CFCC 52587 | *Kadsura longipedunculata* | Beijing, China | MH121440 | MH121480 | MH121522 | MH121564 | MH121601 |
| *Diaporthe kochmanii* | BRIP 54033^T^ | *Helianthus annuus* | Australia | NA | NA | NA | JN645809 | NA |
| *Diaporthe kongii* | BRIP 54031^T^ | *Helianthus annuus* | Australia | NA | NA | NA | NA | KJ197272 |
| *Diaporthe litchicola* | BRIP 54900^T^ | *Litchi chinensis* | Australia | NA | NA | LC041036 | JX862539 | NA |
| *Diaporthe lithocarpus* | CGMCC 3.15175^T^ | *Lithocarpus glabra* | China | KF576235 | NA | KC135104 | KC153095 | KF576311 |
| *Diaporthe longicicola* | CGMCC 3.17089^T^ | *Lithocarpus glabra* | China | NA | NA | KF576267 | KF576242 | KF576291 |
| *Diaporthe longicolla* | FAU599 | *Glycine max* | USA | KJ612124 | KJ659188 | KJ590728 | KJ590767 | KJ610883 |
| *Diaporthe longispora* | CBS 194.36^T^ | *Ribes* sp*.* | Canada | KC343377 | KC343619 | MH855769 | KC343861 | KC344103 |
| *Diaporthe lonicerae* | MFLUCC 17-0963^T^ | *Lonicera* sp. | Italy | KY964116 | NA | KY964190 | KY964146 | KY964073 |
| *Diaporthe lusitanicae* | CBS 123212^T^ | *Foeniculum vulgare* | Portugal | KC343378 | KC343620 | MH863279 | KC343862 | KC344104 |
| *Diaporthe macintoshii* | BRIP 55064a^T^ | *Rapistrum rugosum* | Australia | NA | NA | KJ197289 | KJ197251 | KJ197269 |
| *Diaporthe mahothocarpus* | CGMCC 3.15181 | *Lithocarpus glabra* | China | NA | NA | KC153096 | KC153087 | KF576312 |
| *Diaporthe malorum* | CAA 734 | *Malus domestica* | Portugal | KY435658 | KY435648 | KY435638 | KY435627 | KY435668 |
| *Diaporthe maritima* | DAOM 695742^T^ | *Picea ruben* | Canada | NA | NA | KU552025 | KU552023 | KU574615 |
| *Diaporthe masirevicii* | BRIP 54256 | *Glycine max* | Australia | NA | NA | KJ197277 | KJ197238 | KJ197256 |
| *Diaporthe mayteni* | CBS 133185^T^ | *Maytenus ilicifolia* | Brazil | KC343381 | KC343623 | KC343139 | KC343865 | KC344107 |
| *Diaporthe maytenicola* | CPC 21896^T^ | *Maytenus acuminata* | South Africa | NA | NA | KF777157 | NA | KF777250 |
| *Diaporthe melonis* | CBS 435.87 | *Glycine soja* | Indonesia | KC343383 | KC343625 | KC343141 | KC343867 | KC344109 |
| *Diaporthe middletonii* | BRIP 54884e^T^ | *Rapistrum rugosum* | Australia | NA | NA | KJ197286 | KJ197248 | KJ197266 |
| *Diaporthe miriciae* | BRIP 54736j^T^ | *Helianthus annuus* | Australia | NA | NA | KJ197282 | KJ197244 | KJ197262 |
| *Diaporthe momicola* | MFLUCC 16-0113 | *Prunus persica* | China | NA | KU557611 | KU557563 | KU557631 | KU55758 |
| *Diaporthe multigutullata* | CFCC 53095 | *Citrus maxima* | China | MK442967 | MK442992 | MK432645 | MK578121 | MK578048 |
| *Diaporthe multigutullata* | CFCC 53096 | *Citrus maxima* | China | MK442968 | MK442993 | MK432646 | MK578122 | MK578049 |
| *Diaporthe multigutullata* | ZJUD98 | *Citrus grandis* | China | NA | KJ490575 | KJ490633 | KJ490512 | KJ490454 |
| *Diaporthe musigena* | CBS 129519^T^ | *Musa* sp. | Australia | KC343385 | KC343267 | KC343143 | KC343869 | KC344111 |
| *Diaporthe neilliae* | CBS 144.27^T^ | *Spiraea* sp. | USA | KC343386 | KC343628 | KC343144 | KC343870 | KC344112 |
| *Diaporthe neoarctii* | CBS 109490^T^ | *Ambrosia trifida* | USA | KC343387 | KC343629 | KC343145 | KC343871 | KC344113 |
| *Diaporthe neoraonikayaporum* | MFLUCC 14-1136 | *Tectona grandis* | Thailand | KU749356 | NA | KU712449 | KU749369 | KU743988 |
| *Diaporthe nobilis* | CBS 587.79 | *Pinus parviflora var* | Japan | KC343395 | KC343637 | KC343153 | KC343879 | KC344121 |
| *Diaporthe nothofagi* | BRIP 54801^T^ | *Nothofagus cunninghamii* | Australia | NA | NA | JX862530 | JX862536 | KF170922 |
| *Diaporthe novem* | CBS 127269^T^ | *Glycine max* | Croatia | KC343397 | KC343639 | KC343155 | KC343881 | KC344123 |
| *Diaporthe ocoteae* | CPC 26217^T^ | *Ocotea bullata* | France | NA | NA | KX228293 | NA | KX228388 |
| *Diaporthe oraccinii* | LC3166^T^ | *Camellia sinensis* | China | NA | KP293517 | KP267863 | KP267937 | KP293443 |
| *Diaporthe ovalispora* | ZJUD93 | *Citrus limon* | China | NA | KJ490570 | KJ490628 | KJ490507 | KJ490449 |
| *Diaporthe ovoicicola* | CGMCC 3.17093 | *Lithocarpus glabra* | China | KF576223 | NA | KF576265 | KF576240 | KF576289 |
| *Diaporthe oxe* | CBS 133186^T^ | *Maytenus ilicifolia* | Brazil | KC343406 | KC343648 | KC343164 | KC343890 | KC344132 |
| *Diaporthe padina* | CFCC 52590^T^ | *Padus racemosa* | China | MH121443 | MH121483 | MH121525 | MH121567 | MH121604 |
| *Diaporthe padina* | CFCC 52591 | *Padus racemosa* | China | MH121444 | MH121484 | MH121526 | MH121568 | MH121605 |
| *Diaporthe pandanicola* | MFLUCC 17-0607 | Pandanaceae | Thailand | NA | NA | MG646974 | NA | MG646930 |
| *Diaporthe paranensis* | CBS 133184^T^ | *Maytenus ilicifolia* | Brazil | KC343413 | KC343655 | KC343171 | KC343897 | KC344139 |
| *Diaporthe parapterocarpi* | CPC 22729 | *Pterocarpus brenanii* | Zambia | NA | NA | KJ869138 | NA | KJ869248 |
| *Diaporthe pascoei* | BRIP 54847^T^ | *Persea americana* | Australia | NA | NA | MK111097 | JX862538 | KF170924 |
| *Diaporthe passiflorae* | CPC 19183 | *Passiflora edulis* | Netherlands | NA | NA | JX069860 | NA | NA |
| *Diaporthe passifloricola* | CPC 27480^T^ | *Passiflora foetida* | Malaysia | NA | KX228367 | KX228292 | NA | KX228387 |
| *Diaporthe penetriteum* | LC3215 | *Camellia sinensis* | China | NA | NA | KP267879 | KP293532 | KP267953 |
| *Diaporthe perjuncta* | CBS 109745^T^ | *Ulmus glabra* | Austria | KC343414 | KC343656 | KC343172 | KC343898 | KC344140 |
| *Diaporthe perseae* | CBS 151.73 | *Persea gratissima* | Netherlands | KC343415 | NA | KC343173 | NA | NA |
| *Diaporthe pescicola* | MFLUCC 16-0105 | *Prunus persica* | China | KU557603 | NA | KU557555 | KY400831 | KU557579 |
| *Diaporthe phaseolorum* | AR4203^T^ | *Phaseolus vulgaris* | USA | KJ612135 | KJ659220 | KJ590738 | KJ590739 | KJ610893 |
| *Diaporthe podocarpi-macrophylli* | LC6155 | *Podocarpus macrophyllus* | Japan | KX999278 | KX999246 | KX986774 | KX999167 | KX999207 |
| *Diaporthe pseudomangiferae* | CBS 101339^T^ | *Mangifera indica* | Dominican Republic | KC343423 | KC343665 | KC343181 | KC343907 | KC344149 |
| *Diaporthe pseudophoenicicola* | CBS 176.77 | *Mangifera indica* | Iraq | KC343425 | KC343667 | KC343183 | KC343909 | KC344151 |
| *Diaporthe pseudotsugae* | MFLU 15-3228^T^ | *Pseudotsuga menziesii* | Italy | KY964138 | NA | KY964225 | KY964181 | KY964108 |
| *Diaporthe psoraleae* | CPC 21634 | *Psoralea pinnata* | South Africa | NA | NA | KF777158 | KF777245 | KF777251 |
| *Diaporthe psoraleae-pinnatae* | CPC 21638^T^ | *Psoralea pinnata* | South Africa | NA | NA | KF777159 | NA | KF777252 |
| *Diaporthe pterocarpicola* | MFLUCC 10-0580a^T^ | *Pterocarpus indicus* | Thailand | JX197433 | NA | JQ619887 | JX275403 | JX275441 |
| *Diaporthe pulla* | CBS 338.89^T^ | *Hedera helix* | Yugoslavia | KC343394 | KC343636 | KC343152 | KC343878 | KC344120 |
| *Diaporthe pyracanthae* | CAA483 | *Pyracantha coccinea* | Portugal | KY435645 | KY435656 | KY435635 | KY435625 | KY435666 |
| *Diaporthe racemosae* | CPC 26646 | *Euclea racemosa* | South Africa | MG600219 | MG600221 | MG600223 | MG600225 | MG600227 |
| *Diaporthe raonikayaporum* | CBS 133182 | *Spondias mombin* | Brazil | KC343430 | KC343672 | KC343188 | KC343914 | KC344156 |
| *Diaporthe ravennica* | MFLUCC 16-0997 | *Clematis vitalba* | Italy | NA | NA | NA | MT394670 | NA |
| *Diaporthe rhusicola* | CPC 18191 | *Rhus pendulina* | South Africa | NA | NA | JF951146 | NA | NA |
| *Diaporthe rosae* | MFLUCC 17-2658 | *Rosa* sp*.* | United Kingdom | MG829273 | NA | MG828894 | NA | MG843878 |
| *Diaporthe rosicola* | MFLU 17-0646^T^ | *Rosa* sp. | United Kingdom | MG829274 | NA | MG828895 | MG829270 | MG843877 |
| *Diaporthe rostrata* | CFCC 50062^T^ | *Juglans mandshurica* | China | KP208849 | KP208851 | KP208847 | KP208853 | KP208855 |
| *Diaporthe rostrata* | CFCC 50063 | *Juglans mandshurica* | China | KP208850 | KP208852 | KP208848 | KP208854 | KP208856 |
| *Diaporthe rudis* | AR3422^T^ | *Laburnum anagyroides* | Austria | KC843146 | NA | KC843331 | KC843090 | KC843177 |
| *Diaporthe saccarata* | CBS 116311^T^ | *Protea repens* | South Africa | KC343432 | KC343674 | KC343190 | KC343916 | KC344158 |
| *Diaporthe sackstonii* | BRIP 54669b^T^ | *Helianthus annuus* | Australia | NA | NA | KJ197287 | KJ197249 | KJ197267 |
| *Diaporthe salicicola* | BRIP 54825^T^ | *Salix purpurea* | Australia | NA | NA | JX862531 | JX862537 | KF170923 |
| *Diaporthe sambucusii* | CFCC 51986^T^ | Sambucus williamsii | Beijing, China | KY852499 | KY852503 | KY852495 | KY852507 | KY852511 |
| *Diaporthe sambucusii* | CFCC 51987 | Sambucus williamsii | Beijing, China | KY852500 | KY852504 | KY852496 | KY852508 | KY852512 |
| *Diaporthe schimae* | CFCC 53103 | Schima superba | China | MK442962 | MK442987 | MK442640 | MK578116 | MK578043 |
| *Diaporthe schimae* | CFCC 53104 | Schima superba | China | MK442963 | MK442988 | MK442641 | MK578117 | MK578044 |
| *Diaporthe schimae* | CFCC 53105 | Schima superba | China | MK442964 | MK442989 | MK442642 | MK578118 | MK578045 |
| *Diaporthe schini* | CBS 133181^T^ | *Schinus terebinthifolius* | Brazil | KC343433 | KC343675 | KC343191 | KC343917 | KC344159 |
| *Diaporthe schisandrae* | CFCC 51988^T^ | *Schisandra chinensis* | China | KY852501 | KY852505 | KY852497 | KY852509 | KY852513 |
| *Diaporthe schisandrae* | CFCC 51989 | *Schisandra chinensis* | China | KY852502 | KY852506 | KY852498 | KY852510 | KY852514 |
| *Diaporthe schoeni* | MFLU 15-1279^T^ | *Schoenus nigricans* | Italy | KY964139 | NA | KY964226 | KY964182 | KY964109 |
| *Diaporthe sclerotioides* | CBS 296.67 | *Cucumis sativus* | Netherlands | KC343435 | KC343677 | MH858974 | KC343919 | KC344161 |
| *Diaporthe sennae* | CFCC 51636^T^ | *Senna bicapsularis* | China | KY228875 | NA | KY203724 | KY228885 | KY228891 |
| *Diaporthe sennae* | CFCC 51637 | *Senna bicapsularis* | China | KY228876 | NA | KY203725 | KY228886 | KY228892 |
| *Diaporthe sennicola* | CFCC 51634^T^ | *Senna bicapsularis* | China | KY228873 | KY228879 | KY203722 | KY228883 | KY228889 |
| *Diaporthe sennicola* | CFCC 51635 | *Senna bicapsularis* | China | KY228874 | KY228880 | KY203723 | KY228884 | KY228890 |
| *Diaporthe serafiniae* | BRIP 55665a^T^ | *Helianthus annuus* | Australia | NA | NA | KJ197274 | KJ197236 | KJ197254 |
| *Diaporthe shaanxiensis* | CFCC 53106 | on branches of liana | Shaanxi, China | MK442976 | MK443001 | MK432654 | MK578130 | NA |
| *Diaporthe shaanxiensis* | CFCC 53107 | on branches of liana | Shaanxi, China | MK432977 | MK432002 | MK432655 | MK578131 | NA |
| *Diaporthe siamensis* | MFLUCC 10-0573a | *Dasymaschalon* sp. | Thailand | JQ619897 | NA | NA | JX275393 | JX275429 |
| *Diaporthe sojae* | FAU635^T^ | *Glycine max* | USA | KJ612116 | KJ659208 | KJ590719 | KJ590762 | KJ610875 |
| *Diaporthe spartinicola* | CPC 24951 | *Spartium junceμm* | Spain | NA | KR857696 | KR611879 | NA | KR857695 |
| *Diaporthe sterilis* | CBS 136969^T^ | Vaccinium corymbosum | Italy | KJ160548 | MF418350 | KJ160579 | KJ160611 | KJ160528 |
| *Diaporthe stictica* | CBS 370.54 | *Buxus sampervirens* | Italy | KC343454 | KC343696 | KC343212 | KC343938 | KC344180 |
| *Diaporthe subclavata* | ZJUD95 | *Citrus unshiu* | China | NA | KJ490572 | KJ490630 | KJ490509 | KJ490451 |
| *Diaporthe subcylindrospora* | KUMCC 17-0151 | dead wood | China | NA | NA | MG746629 | MG746630 | MG746631 |
| *Diaporthe subellipicola* | KUMCC 17-0153 | NA | China | NA | NA | MG746632 | MG746633 | MG746634 |
| *Diaporthe subordinaria* | CBS 464.90 | Plantago lanceolata | South Africa | KC343456 | KC343698 | KC343214 | KC343940 | KC344182 |
| *Diaporthe taoicola* | MFLUCC 16-0117 | Prunus persica | China | NA | NA | KU557567 | KU557636 | KU557591 |
| *Diaporthe tectonae* | MFLUCC 12-0777 | *Tectona grandis* | Thailand | KU749345 | NA | KU712430 | KU749359 | KU743977 |
| *Diaporthe tectonendophytica* | MFLUCC 13-0471 | *Tectona grandis* | Thailand | KU749354 | NA | KU712439 | KU749367 | KU743986 |
| *Diaporthe tectonigena* | MFLUCC 12-0767 | *Camellia sinensis* | China | KX999284 | KX999254 | KX986782 | KX999174 | KX999214 |
| *Diaporthe terebinthifolii* | CBS 133180^T^ | *Schinus terebinthifolius* | Brazil | KC343458 | KC343700 | KC343216 | KC343942 | KC344184 |
| *Diaporthe ternstroemia* | CGMCC 3.15183 | *Ternstroemia gymnanthera* | China | NA | NA | KC153098 | KC153089 | NA |
| *Diaporthe thunbergii* | MFLUCC 10-0576a^T^ | *Thunbergia laurifolia* | Thailand | JX197440 | NA | JQ619893 | JX275409 | NA |
| *Diaporthe thunbergiicola* | MFLUCC 12-0033^T^ | *Thunbergia laurifolia* | Thailand | NA | NA | KP715097 | KP715098 | NA |
| *Diaporthe tibetensis* | CFCC 51999^T^ | *Juglandis regia* | China | MF279888 | MF279828 | MF279843 | MF279858 | MF279873 |
| *Diaporthe tibetensis* | CFCC 52000 | *Juglandis regia* | China | MF279889 | MF279829 | MF279844 | MF279859 | MF279874 |
| *Diaporthe torilicola* | MFLUCC 17-1051^T^ | Torilis arvensis | Italy | KY964127 | NA | KY964212 | KY964168 | KY964096 |
| *Diaporthe toxica* | CBS 534.93^T^ | Lupinus angustifolius | Australia | KC343462 | KC343704 | KC343220 | KC343946 | KC344188 |
| *Diaporthe tulliensis* | BRIP 62248a | Theobroma cacao | Australia | NA | NA | KR936130 | KR936133 | KR936132 |
| *Diaporthe ueckerae* | FAU656^T^ | *Cucumis melo* | USA | KJ612122 | KJ659215 | KJ590726 | KJ590747 | KJ610881 |
| *Diaporthe undulata* | LC6624 | NA | China | NA | KX999269 | KX986798 | KX999190 | KX999230 |
| *Diaporthe unshiuensis* | ZJUD52 | Citrus unshiu | Zhejiang, China | NA | KJ490529 | KJ490587 | KJ490466 | KJ490408 |
| *Diaporthe vaccinii* | CBS 160.32^T^ | *Oxycoccus macrocarpos* | USA | MH121426 | MH121462 | MH121502 | MH121544 | MH121584 |
| *Diaporthe vangueriae* | CBS 137985^T^ | *Vangueria infausta* | Zambia | NA | NA | KJ869137 | NA | KJ869247 |
| *Diaporthe vawdreyi* | BRIP 57887a | *Psidium guajava* | Australia | NA | NA | KR936126 | KR936129 | KR936128 |
| *Diaporthe velutina* | LC4421 | *Neolitsea* sp. | China | NA | KX999261 | KX986790 | KX999182 | KX999223 |
| *Diaporthe verniciicola* | CFCC 53109 | *Vernicia montana* | China | MK574583 | MK574599 | MK573944 | MK574619 | MK574639 |
| *Diaporthe verniciicola* | CFCC 53110 | *Vernicia montana* | China | MK574584 | MK574600 | MK573945 | MK574620 | MK574640 |
| *Diaporthe virgiliae* | CMW 40748 | *Virgilia oroboides* | South Africa | NA | NA | KP247556 | NA | KP247575 |
| *Diaporthe xishuangbanica* | LC6707 | *Camellia sinensis* | China | NA | KX999255 | KX986783 | KX999175 | KX999216 |
| *Diaporthe xunwuensis* | CFCC 53085 | Unknown dead wood | China | MK442983 | MK443008 | MK432663 | MK578137 | MK578063 |
| *Diaporthe xunwuensis* | CFCC 53086 | Unknown dead wood | China | MK442984 | MK443009 | MK432664 | MK578138 | MK578064 |
| *Diaporthe yunnanensis* | LC6168 | NA | China | KX999290 | KX999267 | KX986796 | KX999188 | KX999228 |
| *Diaporthe ukurunduensis* | CFCC 52592^T^ | *Acer ukurunduense* | China | MH121445 | MH121485 | MH121527 | MH121569 | NA |
| *Diaporthe ukurunduensis* | CFCC 52593 | *Acer ukurunduense* | China | MH121446 | MH121486 | MH121528 | MH121570 | NA |
| *Diaporthe unshiuensis* | CFCC 52594 | *Carya illinoensis* | China | MH121447 | MH121487 | MH121529 | MH121571 | MH121606 |
| *Diaporthe unshiuensis* | CFCC 52595 | *Carya illinoensis* | China | MH121448 | MH121488 | MH121530 | MH121572 | MH121607 |
| *Diaporthella corylina* | CBS 121124 | *Corylus* sp*.* | China | KC343246 | KC343488 | KC343004 | KC343730 | KC343972 |

^2^ ATCC: American Type Culture Collection, Virginia, USA; AR, DP, FAU isolates in culture collection of Systematic Mycology and Microbiology Laboratory, USDA-ARS, Beltsville, Maryland, USA; BRIP: Australian plant pathogen culture collection, Queensland, Australia; CBS: Westerdijk Fungal Biodiversity Institute, Utrecht, The Netherlands; CFCC: China Forestry Culture Collection Center, China; CGMCC: China General Microbiological Culture Collection; CMW: culture collection (CMW) of the Forestry and Agricultural Biotechnology Institute; ICMP: International Collection of Microorganisms from Plants, Landcare Research, Auckland, New Zealand; LC: working collection of Lei Cai, housed at Institute of Microbiology, CAS, China; MFLUCC: Mae Fah Luang University Culture Collection; ZJUD: Zhe Jiang University, China. All the *Diaporthe* species listed from *Corylus* plants are marked with *.
