# Supplementary material for: Cytospora and Diaporthe Species Associated With Hazelnut Canker and Dieback in Beijing, China
Source: Front Cell Infect Microbiol. 2021 Aug 2;11:664366. doi: 10.3389/fcimb.2021.664366 (PMC8366500; doi:10.3389/fcimb.2021.664366)
Supplement: Supplementary file 4 [file Table_4.docx]

**Supplymentary Table 4.** Isolation of fungi pathogens from infected hazelnut branches.

| **Species** | **Strains number** | **Number of each species/total number (%)** |
| --- | --- | --- |
| *Cytospora curvispora* | 6 | 6/51 (11.76) |
| *Cytospora corylina* | 4 | 4/51 (7.84) |
| *Cytospora leucostoma* | 4 | 4/51 (7.84) |
| *Diporthe corylicola* | 33 | 33/51 (64.71) |
| *Diaporthe eres* | 4 | 4/51 (7.84) |
